# Trophic diversity in the evolution and community assembly of loricariid catfishes

**DOI:** 10.1186/1471-2148-12-124

**Published:** 2012-07-26

**Authors:** Nathan K Lujan, Kirk O Winemiller, Jonathan W Armbruster

**Affiliations:** 1 Department of Wildlife and Fisheries Sciences, Texas A&M University, College Station, TX, 77843, USA; 2 Department of Biological Sciences, Auburn University, Auburn, AL, 36849, USA; 3 Department of Natural History, Royal Ontario Museum, Toronto, ON, M5S 2C6, Canada

## Abstract

**Background:**

The Neotropical catfish family Loricariidae contains over 830 species that display extraordinary variation in jaw morphologies but nonetheless reveal little interspecific variation from a generalized diet of detritus and algae. To investigate this paradox, we collected δ^13^C and δ^15^N stable isotope signatures from 649 specimens representing 32 loricariid genera and 82 species from 19 local assemblages distributed across South America. We calculated vectors representing the distance and direction of each specimen relative to the δ^15^N/δ^13^C centroid for its local assemblage, and then examined the evolutionary diversification of loricariids across assemblage isotope niche space by regressing the mean vector for each genus in each assemblage onto a phylogeny reconstructed from osteological characters.

**Results:**

Loricariids displayed a total range of δ^15^N assemblage centroid deviation spanning 4.9‰, which is within the tissue–diet discrimination range known for Loricariidae, indicating that they feed at a similar trophic level and that δ^15^N largely reflects differences in their dietary protein content. Total range of δ^13^C deviation spanned 7.4‰, which is less than the minimum range reported for neotropical river fish communities, suggesting that loricariids selectively assimilate a restricted subset of the full basal resource spectrum available to fishes. Phylogenetic regression of assemblage centroid-standardized vectors for δ^15^N and δ^13^C revealed that loricariid genera with allopatric distributions in disjunct river basins partition basal resources in an evolutionarily conserved manner concordant with patterns of jaw morphological specialization and with evolutionary diversification via ecological radiation.

**Conclusions:**

Trophic partitioning along elemental/nutritional gradients may provide an important mechanism of dietary segregation and evolutionary diversification among loricariids and perhaps other taxonomic groups of apparently generalist detritivores and herbivores. Evolutionary patterns among the Loricariidae show a high degree of trophic niche conservatism, indicating that evolutionary lineage affiliation can be a strong predictor of how basal consumers segregate trophic niche space.

## Background

**Figure 1:**
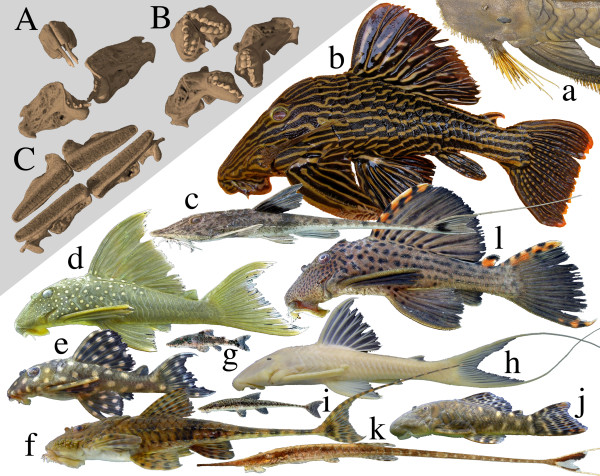
**Representative morphological diversity within Loricariidae.** Inset: CT reconstructions of upper and lower jaws of (**A**) *Leporacanthicus* (an insectivore); (**B**) *Panaque* (a wood-eater); and (**C**) *Chaetostoma* (a detritivore-algivore). Photos (scaled to approximate relative size): (**a**) *Pseudancistrus pectegenitor* (Ancistrini), (**b**) *Panaque armbrusteri* (Ancistrini), (**c**) *Pseudohemiodon* sp. (Loricariini), (**d**) *Hemiancistrus subviridis* (Ancistrini), (**e**) *Hypancistrus contradens* (Ancistrini), (**f**) *Spatuloricaria* sp. (Loricariini), (**g**) *Parotocinclus eppleyi* (Hypoptopomatinae), (**h**) *Hemiancistrus pankimpuju* (Ancistrini), (**i**) *Oxyropsis acutirostra* (Hypoptopomatinae), (**j**) *Chaetostoma* sp. (Ancistrini), (**k**) *Farlowella* sp. (Farlowellini), (**l**) *Leporacanthicus triactis* (Ancistrini). Fish photos by N. K. Lujan (*b*,*g*,*h*,*i*,*j*,*k*) and M. H. Sabaj Pérez (*a*,*c*,*d*,*e*,*f*,*l*).

In rivers of tropical South America, loricariid catfishes (also known as plecos or suckermouth armored catfishes) are ubiquitous and easily identified by their distinctive armored plating and ventrally positioned jaws with a fleshy oral disk. Loricariid jaws permit efficient foraging on benthic food items and span a wide range of morphologies, from robust jaws specialized for gouging wood [1-4]; Figure 1B, to gracile jaws for winnowing loose sediments, and pincer-like jaws for probing crevices [1-3]; Figure 1A. Despite their taxonomic richness and jaw diversity, loricariids display relatively little dietary diversity. We reviewed published studies of loricariid gut contents spanning over 100 species, 20 drainages, and 13 countries (Additional file 1: Table S1). Findings from these studies reveal diets dominated by fine particulate detritus, usually of undetermined origin, mixed with lesser fractions of algae, other plant matter, and occasionally benthic invertebrates.

Detritus is the principle pathway by which primary production enters food webs globally [5]. In rivers, detritus is foundational to metazoan food webs [6] and critical to ecosystem function [7]. This is especially true in tropical rivers, where detritivorous fishes are highly diverse and abundant enough to support major commercial fisheries [8]. Unlike predators, which commonly select prey based on their general morphology [9], detritivores selectively forage based on cryptic and continuously variable aspects of the elemental and nutritional composition of detritus [1,10-12]. Although presumably detectable by detritivores [10,11], variation in detrital quality is difficult to quantify and remains poorly understood. The extent to which dietary discrimination yields niche partitioning within local detritivore assemblages and the influence of phylogeny on partitioning of detritivore trophic niches are just beginning to be examined [1,13].

A growing body of theoretical [12] and empirical [14,15] research suggests that diverse, sympatric, and apparently functionally redundant assemblages of herbivores may coexist via partitioning of relatively cryptic elemental and nutritional gradients of the food resource spectrum. Sympatric herbivorous insects partition trophic niches according to ratios of carbohydrate and protein [14], and apparently non-selective, filter-feeding bivalves demonstrate similarly cryptic niche differentiation along stoichiometric gradients [15]. Taxonomic groupings observed in these and other studies [16,17] suggest that phylogeny strongly influences the biochemical composition of these consumers’ diets; however, modern phylogenetic comparative methods have yet to be applied to investigations of niche partitioning among detritivores. Herein, we examine the evolutionary context of trophic niche diversity among detritivorous loricariids by estimating trophic positions of individuals in local assemblage isotope space, then regressing these data onto the most recent and comprehensive phylogeny for the family.

Stable isotope analysis provides a powerful means for investigating trophic ecology [1,8,9,15], in part because it can more effectively reveal trophic partitioning among detritivores than gut contents analysis [1]. Detritivores have rapid gut passage rates [10], derive major nutritional contributions from microbial decomposers [1,18], and selectively feed upon heterogeneous yet amorphous detrital fractions [10,11]. These features reduce the utility of visual analysis of gut contents, but lend themselves to isotope analyses capable of quantifying and integrating food items assimilated over periods spanning several days to weeks [19]. Consumer tissue δ^13^C reflects an average aggregate isotopic signature of all basal production sources assimilated [19], and δ^15^N corresponds to variation in trophic level and dietary protein content [20]. Studies inferring community trophic structure from isotopic data often lack precision in differentiating basal resource categories [21,22]; however, even without finely resolved basal resource data, distributions of consumers in isotope biplot space can be used to examine trophic niche size, spacing in community niche space, resource partitioning [23], and niche shifts in time or space [24,25].

Herein, we introduce a novel method that builds upon several recently proposed approaches for comparing community trophic structure using the isotopic centroid (the mean for all species in a local assemblage) as a reference point [23-25]. Instead of calculating only distances from the centroid to component taxa [23] or patterns of centroid movement through absolute isotope space [24,25], we use vectors to describe both distance and position of consumer taxa relative to their local assemblage centroid, and use the centroid as a means of standardization that allows datasets from different times and places to be combined. Assemblage centroid-standardized isotope vector analysis (ACSIVA) produces a metric that describes an individual consumer’s trophic position in relation to other consumers in the same habitat (in contrast to the traditional practice of presenting isotopic ratios of consumers in relation to either resources or an arbitrary isotopic standard [1,19-22]). ACSIVA facilitates comparisons across geographic and temporal ranges as well as phylogenetic regressions to reveal evolutionary influence on contemporary assemblage structure.

Given that loricariid diets are largely limited to detritus, algae, and in special cases other basal resources such as wood [1] (see Additional file 1: Table S1 for summary of published diet studies), we predicted that the total range of species δ^15^N centroid deviations would reflect a single trophic level (i.e., this range will be less than the 5.2‰ maximum tissue–diet discrimination range reported for Loricariidae [19]). Also, given recent research [1] suggesting that loricariids with derived jaw morphologies [2] defined by narrow, medial clusters of elongate teeth (e.g., Figure 1A) could use these teeth to supplement their detritivorous diet with insect larvae pried from holes in benthic substrates (Additional file 1: Table S1), we predicted that taxa with these jaws will be ^15^ N-enriched relative to loricariids with broader jaws and greater numbers of shorter teeth designed for scraping or raking (e.g., Figure 1C). Finally, given the potential that dietary enrichment with insect protein may correspond with specialized morphologies or behaviors [1,2], and that vertical (δ^15^N) trophic position can likely be maintained across food chains supported by primary production sources with different δ^13^C signatures [20-23], we predicted that the relative position of loricariids along the δ^15^N axis should be more phylogenetically conserved than distributions across the δ^13^C axis.

## Results

**Figure 2:**
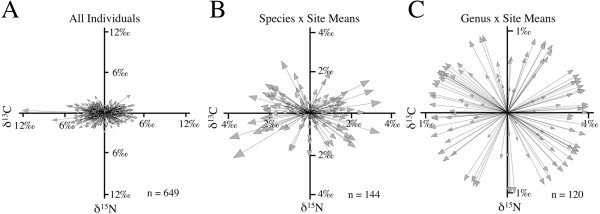
**The total sample space (A) consisting of 649 vectors representing the distance and direction to individual isotope samples from the mean species centroid in δ**^**15**^**N/δ**^**13**^**C isotope space at 19 localities.** Mean vectors representing partitions of the data set according to taxon identity at the rank of species **(B)** and genus **(C)** by site.

Mean values for loricariid species displayed a total range of δ^15^N assemblage centroid deviation spanning 4.9‰ and a total range of δ^13^C deviation spanning 7.4‰ (Figure 2B). As predicted, total δ^15^N range fell within the tissue–diet discrimination range (i.e., trophic fractionation range), observed in a controlled laboratory study of Loricariidae (4.1–5.2‰) [19], suggesting that loricariids essentially occupy a single trophic level within which δ^15^N variation corresponds to differences in dietary protein content [20]. Total δ^13^C range of species means was less than the distribution of δ^13^C ranges (10–17.5‰) reported for entire fish communities in four different Neotropical rivers [22], suggesting that loricariids selectively consume and assimilate a restricted subset of the basal production spectrum supporting fish biomass in these ecosystems.

**Figure 3:**
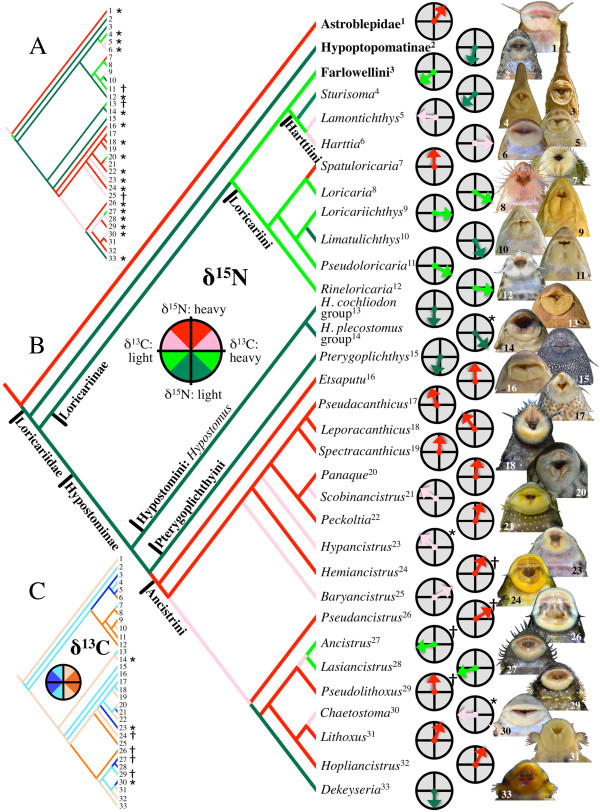
**Evolutionary patterns of loricariid trophic diversification revealed by assemblage centroid-standardized isotope vector analysis (ACSIVA) of C and N stable isotope data (Figure**3**; See Additional file****4****: Figures 3–10 for isotope biplots of all local assemblages examined).** Cladograms **A** and **B** represent the hypothesized ancestral distributions of lineages along a vertical axis of ^15^ N-enrichment relative to assemblage centroid (pink to red being ^15^ N-enriched relative to centroid, light green to green being ^15^ N-depleted relative to centroid). Cladogram A represents a phylogenetic regression of 649 individual vectors (Figure 3A) grouped by genus irrespective of site (see Additional file 2: Figure S1 for a full size version of this phylogeny), and cladogram B represents a regression of 120 mean genus x site vectors (Figure 3 C). Circle plots illustrate the direction of mean genus x site vectors for each genus and statistical significance of the mean vector is indicated by * (Rayleigh’s Z test: *P* < 0.05) or † (0.10 > *P* > 0.05; Tables 1, 2). Cladogram **C** resulted from the same analysis as B, but is color-coded to reflect hypothesized distributions of ancestral lineages along a horizontal axis of ^13^ C-enrichment relative to assemblage centroid (see Additional file 3: Figure S2 for a full size version of this phylogeny). Oral disk photos by N. K. Lujan or M. H. Sabaj Pérez.

**Figure 4:**
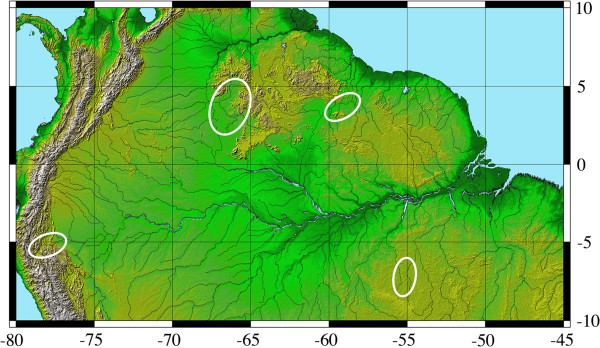
**Map showing the distributions across northern South America of four regions (ovals) across which 19 local loricariid assemblages were sampled (See Additional file****5****: Table S2 for list of species by locality and region, Additional file****4****: Figures S3–10 for isotope biplots of all local assemblages)**.

Phylogenetic regressions were conducted first on the entire data set of 649 individual assemblage centroid-standardized vectors (Figure 2A, Table 1). Because of the highly uneven distribution of sample sizes across sites and the potential for site bias, a second analysis was conducted on a more restricted data set consisting of 120 vectors representing the mean trophic niche of each genus at each site (Figure 2B, Table 2). In each case, the phylogenetic regression took full consideration of the two-dimensional (X = δ^13^C, Y = δ^15^N) character of the data but for interpretational clarity, cladograms color-coded to represent evolutionary shifts along the vertical δ^15^N axis (Figure 3A, B, Additional file 2: Figure S1) are presented separately from cladograms representing variation along the horizontal δ^13^C axis (Figure 4C, Additional file 3: Figure S2). In the analysis of individual vectors, 20 of 33 genera had a mean vector significant at *P* < 0.1 (Figure 3A, Table 1). In the analysis of genus mean vectors, the number of genera with statistically significant mean vectors dropped to seven (Figure 3B, Table 2), largely because many loricariid genera have geographically restricted ranges and most were represented in this study at only one or few sites (Table 1). Regardless, the evolutionary patterns recovered by these analyses were nearly identical (Figure 3A, B). Both recovered a basal division between ^15^ N-enriched Astroblepidae and ^15^ N-depleted Loricariidae, which is consistent with gut contents data describing Astroblepidae as predominantly insectivorous and Loricariidae as predominantly herbivorous-detritivorous (See Additional file 1: Table S1 for summary of published diet data). Within Loricariidae, both analyses also revealed seven transitions between relatively ^15^ N-depleted and relatively ^15^ N-enriched diets, however the locations (nodes) of transition differed slightly (Figure 3A, B). Both analyses also recovered a phylogenetically conserved reversion to ^15^ N-enriched diets at the base of the highly diverse tribe Ancistrini, with only three of 18 ancistrin genera reverting back to relatively ^15^ N-depleted diets (*Ancistrus*, *Dekeyseria*, and *Panaque* in the first analysis, Figure 3A; *Ancistrus*, *Dekeyseria,* and *Lasiancistrus* in the second analysis, Figure 3B).

**Table 1:** **Numbers of individuals and assemblages sampled for each genus, with data and statistics on individual-based vector means for each genus (Additional file ****2****: Figure S1)**

**Genus**	**Individuals**	**Assemblages**	**Mean vector (μ)**	**Length of mean vector (r)**	**Concentration**	**Circular variance**	**Circular standard deviation**	**Rayleigh's Z (*P*)**	**Rao's spacing test (*P*)**
Astroblepidae									
Astroblepus*	6	3	52°	0.74	1.27	0.26	45°	0.031	< 0.05
Loricariidae									
Hypoptopomatinae									
*Hypoptopoma*	7	2	274°	0.22	0.00	0.78	99°	0.722	0.90 > *P* > 0.50
Hypostominae									
Ancistrini									
Ancistrus*	44	9	192°	0.45	0.99	0.56	73°	0.000	< 0.01
*Baryancistrus*†	23	4	14°	0.32	0.67	0.68	87°	0.098	< 0.01
Chaetostoma*	44	5	179°	0.68	1.88	0.32	51°	0.000	< 0.01
*Dekeyseria*	2	1	271°	1.00	20.46	0.00	4°	0.139	–
Etsaputu*	12	2	120°	0.59	1.36	0.41	59°	0.012	< 0.01
Hemiancistrus*	51	5	60°	0.46	1.03	0.54	72°	0.000	< 0.01
*Hopliancistrus*	5	1	86°	0.31	0.02	0.69	88°	0.650	0.50 > *P* > 0.10
Hypancistrus*	25	4	157°	0.88	4.62	0.12	28°	0.000	< 0.01
Lasiancistrus*	17	4	174°	0.57	1.38	0.43	61°	0.003	< 0.05
Leporacanthicus*	12	2	136°	0.68	1.82	0.32	50°	0.002	< 0.01
*Lithoxus*	2	1	69°	0.97	1.90	0.03	13°	0.158	–
Panaque*	41	5	343°	0.34	0.72	0.66	84°	0.008	< 0.01
Peckoltia*	15	5	113°	0.64	1.59	0.36	55°	0.001	< 0.01
*Pseudacanthicus*	1	1	110°	1.00	–	–	–	0.512	–
Pseudancistrus*	32	4	53°	0.75	2.34	0.25	44°	0.000	< 0.01
Pseudolithoxus*	24	3	108°	0.50	1.16	0.50	67°	0.002	0.10 > *P* > 0.05
*Scobinancistrus*	2	1	149°	0.99	6.29	0.01	7°	0.143	–
*Spectracanthicus*	4	1	86°	0.69	1.72	0.31	49°	0.148	0.50 > *P* > 0.10
Hypostomini									
*H. cochliodon* grp.†	30	7	329°	0.30	0.63	0.70	89°	0.063	< 0.05
*H. plecostomus* grp.*	97	14	302°	0.19	0.39	0.81	104°	0.028	< 0.05
Pterygoplichthyini									
*Pterygoplichthys*	2	2	254°	0.73	0.22	0.27	45°	0.396	–
Loricariinae									
Farlowellini									
*Farlowella*	4	3	251°	0.51	0.77	0.49	66°	0.376	0.50 > *P* > 0.10
Harttiini									
Harttia*	14	2	1°	0.98	18.40	0.02	12°	0.000	< 0.01
Lamontichthys*	10	1	174°	0.97	12.64	0.03	14°	0.000	< 0.01
Sturisoma*	16	2	187°	0.65	1.74	0.35	53°	0.001	< 0.01
Loricariini									
*Limatulichthys*	20	3	304°	0.25	0.51	0.75	96°	0.299	< 0.01
*Loricaria*	10	5	339°	0.36	0.50	0.64	82°	0.289	0.90 > *P* > 0.50
*Loricariichthys*	5	2	6°	0.55	1.01	0.45	63°	0.231	0.50 > *P* > 0.10
*Pseudoloricaria*†	3	1	331°	0.93	2.01	0.07	22°	0.062	–
Rineloricaria*	52	5	313°	0.26	0.54	0.74	94°	0.030	< 0.01
*Spatuloricaria*	17	6	90°	0.21	0.42	0.80	102°	0.497	< 0.01

Significance of mean vectors determined by Rayleigh's Z and uniformity of vector distribution measured by Rao's Spacing test. Taxa with significant mean vectors designated with * (*P* < 0.05) or † (0.10 > *P* > 0.05). Total number of vectors = 649 (Figure 3A).

**Table 2:** **Genus x site mean vector (Figure **4**B) statistics for each genus**

**Genus**	**Mean vector (μ)**	**Length of mean vector (r)**	**Concentration**	**Circular variance**	**Circular standard deviation**	**Rayleigh's Z (*P*)**	**Rao's spacing test (*P*)**
Astroblepidae							
*Astroblepus*	58°	0.83	0.86	0.17	35°	0.128	–
Loricariidae							
Hypoptopomatinae							
*Hypoptopoma*	254°	0.34	0.00	0.66	85°	0.837	–
Hypostominae							
Ancistrini							
*Ancistrus*†	189°	0.53	1.05	0.47	65°	0.08	0.50 > *P* > 0.10
*Baryancistrus*	28°	0.44	0.48	0.56	73°	0.487	0.90 > *P* > 0.50
Chaetostoma*	180°	0.88	2.54	0.12	29°	0.004	< 0.01
*Dekeyseria*	271°	1.00	–	–	–	0.512	–
*Etsaputu*	96°	0.74	0.23	0.26	45°	0.392	–
*Hemiancistrus*†	62°	0.68	1.68	0.32	50°	0.094	0.50 > *P* > 0.10
*Hopliancistrus*	63°	0.31	0.00	0.69	88°	0.864	–
Hypancistrus*	141°	0.93	2.79	0.07	22°	0.02	< 0.05
*Lasiancistrus*	194°	0.46	0.55	0.54	71°	0.458	0.50 > *P* > 0.10
*Leporacanthicus*	128°	0.92	0.66	0.08	23°	0.202	–
*Lithoxus*	70°	1.00	–	–	–	0.512	–
*Panaque*	84°	0.53	0.92	0.47	65°	0.258	0.50 > *P* > 0.10
*Peckoltia*	76°	0.46	0.66	0.54	71°	0.362	0.50 > *P* > 0.10
*Pseudacanthicus*	110°	1.00	–	–	–	0.512	–
*Pseudancistrus*†	36°	0.76	0.98	0.24	42°	0.093	0.10 > *P* > 0.05
*Pseudolithoxus*†	94°	0.94	2.44	0.06	20°	0.056	–
*Scobinancistrus*	149°	1.00	–	–	–	0.512	–
*Spectracanthicus*	86°	1.00	–	–	–	0.512	–
Hypostomini							
*H. cochliodon* grp.	263°	0.20	0.00	0.80	103°	0.713	0.99 > *P* > 0.95
*H. plecostomus* grp.*	307°	0.60	1.41	0.40	58°	0.005	< 0.01
Pterygoplichthyini							
*Pterygoplichthys*	254°	0.73	0.22	0.27	45°	0.399	–
Loricariinae							
Farlowellini							
*Farlowella*	222°	0.54	0.76	0.46	64°	0.459	–
Harttiini							
*Harttia*	2°	1.00	152.99	0.00	1°	0.137	–
*Lamontichthys*	174°	1.00	–	–	–	0.512	–
*Sturisoma*	227°	0.70	0.20	0.30	48°	0.432	–
Loricariini							
*Limatulichthys*	294°	0.83	0.89	0.17	35°	0.124	–
*Loricaria*	331°	0.66	1.56	0.34	52°	0.111	0.10 > *P* > 0.05
*Loricariichthys*	356°	0.89	0.49	0.11	27°	0.229	–
*Pseudoloricaria*	331°	1.00	–	–	–	0.512	–
*Rineloricaria*	354°	0.51	0.84	0.49	67°	0.289	0.10 > *P* > 0.05
*Spatuloricaria*	93°	0.24	0.00	0.76	97°	0.732	0.10 > *P* > 0.05

Significance of mean vectors determined by Rayleigh's Z and uniformity of vector distribution measured by Rao's Spacing test. Taxa with significant mean vectors designated with * (*P* < 0.05) or † (0.10 > *P* > 0.05). Total number of vectors = 120 (Figure 3C).

The only ancistrin exception to be recovered in either analysis as both ^15^ N-depleted and ^13^ C-enriched, was *Panaque*, one of only two extant fish lineages– both in the Loricariidae – known to specialize on a diet consisting almost entirely of wood [1-4]. The other wood-eating lineage is the *Hypostomus cochliodon* group (tribe Hypostomini), a clade that has converged evolutionarily on a *Panaque*-like jaw morphology [1-3] and was also recovered in the individual analysis as having an isotope vector nearly parallel to that of *Panaque* (see Additional file 2 for mean genus vectors from the individual analysis). Moreover, vectors derived from both the individual mean and genus mean for the Loricariinae genera *Loricaria* and *Pseudoloricaria* were ^15^ N-depleted and ^13^ C-enriched, and these genera have also been reported to consume fractions of allochthonous plant material (e.g., seeds, fruits, leaves, and flowers) in addition to detritus (see Additional file 1: Table S1). Although these parallelisms provide evidence that vector direction corresponds with dietary niche, the pattern was only partially corroborated in our reduced analysis of mean genus vectors (Figure 3B), perhaps due to insufficient sample size. Likewise, jaw morphologies among non wood-eating Ancistrini displayed gross correspondence with patterns of ^15^ N-enrichment that were most apparent in the individual analysis (Additional file 2: Figure S1). Genera for which fewer, larger, and more medially clustered teeth (Figure 1A) are taxonomically diagnostic (e.g., *Pseudacanthicus*, *Leporacanthicus*, *Spectracanthicus*, *Scobinancistrus*, *Lithoxus*) tended to be more ^15^ N-enriched than genera with long rows of many small teeth (Figure 1C; e.g., *Ancistrus*, *Baryancistrus*, *Lasiancistrus*, *Chaetostoma*, *Dekeyseria*; Additional file 2: Figure S1) [1-3].

Evolutionary patterns of ^13^ C assimilation were different from those of ^15^ N. Phylogenetic regression of genus mean vectors computed from individual values recovered 11 transitions between relatively ^13^ C-enriched and relatively ^13^ C-depleted lorcariids (Additional file 3: Figure S2), and regression of genus vectors computed from their means at each site recovered 14 transitions (Figure 3C; versus seven transitions for δ^15^N recovered in both analyses). These discrepancies support our hypothesis that loricariid catfishes are more evolutionarily flexible with regard to specific primary production sources consumed compared with the protein content of their diet. Nonetheless, δ^13^C distributions of loricariids also show some evidence of trophic specialization.

Most neotropical fish communities, including loricariid assemblages, avoid consuming or assimilating ^13^ C-enriched C_4_ grasses and selectively consume and assimilate larger fractions of relatively ^13^ C-depleted C_3_ macrophytes, algae, and particulate organic carbon [1,8,22,26]. This may explain the much narrower total range of loricariid mean species δ^13^C centroid deviation (Figure 2B) relative to published ranges of neotropical river fish communities containing diverse species representing multiple families and ecological strategies [22]. Moreover, this subset of basal resources that are preferentially consumed shows relatively broad but consistent patterns of δ^13^C variation that can be used to inform our understanding of trophic diversification and specialization among loricariid lineages. Particulate organic carbon is typically most ^13^ C-depleted, C_3_ macrophytes are most ^13^ C-enriched, and algae, although showing large variation in time and space, tend to have intermediate δ^13^C signatures [22]. Phylogenetic regressions based on the individual-based genus means and site-based genus means revealed similar evolutionary patterns among the Loricariinae (Figure 3C; Additional file 3: Figure 2): Harttiini plus Farlowellini and their common ancestor consume and assimilate relatively ^13^ C-depleted resources (e.g., particulate organic carbon), whereas their sister clade (Loricariini) specializes on relatively ^13^ C-enriched resources (e.g., macrophytes). Both data sets also indicate that the genus *Harttia* underwent a reversion to a relatively ^13^ C-enriched diet. These patterns are corroborated by both gut contents and jaw functional morphological data. Gut contents reveal large fractions of higher plants and plant parts in diets of the Loricariini genera *Loricaria, Pseudoloricaria*, and *Rineloricaria* (whereas *Sturisoma* and *Farlowella* feed mostly on amorphous detritus; Additional file 1: Table S1), and Loricariini occupy a unique, non-overlapping region of jaw functional morphospace distinct from all other loricariids [2].

## Discussion

Evolutionary diversification of Loricariidae in assemblage isotope-niche space demonstrates the potential for herbivores and detritivores to radiate adaptively by partitioning seemingly homogenous food resources along elemental-nutritional gradients. Phylogenetic analyses of both individual and grouped (genus x site mean) data partitions revealed concordant patterns of early diversification into opposite ends of a gradient in dietary protein content as inferred from relative ^15^ N enrichment (Figure 3A, B). In contrast to its sister lineage (Astroblepidae), Loricariidae was recovered as plesiomorphically ^15^ N-depleted, reflecting an evolutionary transition to a mostly herbivorous-detritivorous diet contemporaneous with the origin of a major functional innovation of the jaw that is synapomorphic for Loricariidae: a novel adductor mandibulae division that inserts directly onto the upper jaw [27]. Within Loricariidae, the basal lineages (Hypoptopomatinae, Loricariinae, Hypostomini, and Pterygoplichthyini) were recovered as plesiomorphically ^15^ N-depleted, whereas Ancistrini was revealed to have undergone a derived shift back toward ^15^ N-enriched diets. ^15^ N-enrichment of Ancistrini combined with its nested phylogenetic position suggests that members of this clade may have specializations allowing them to consume basal resources with more protein [20], indicative of a higher quality diet.

Several concordant aspects of Ancistrini morphology, behavior, ecology, and biogeography point to a mechanism by which community ecological processes may have contributed to observed evolutionary patterns in δ^15^N. Ancistrins are distinguished by having well-developed lateral clusters of quill-like cheek-spines (odontodes; Figure 1a) [3,28] that are forcefully erected and used as weapons during inter- and intra-specific threat displays. In aquaria, these threat displays are particularly associated with competition for food and territory (NKL, KOW, JWA, pers. obs.). In natural habitats, ancistrins are known to display remarkably even spacing along the stream bed [29], and biogeographical research [30] has observed that phylogenetically basal, non-ancistrin loricariids are often limited to regions occupied by few or no ancistrins. Together with observed patterns of ^15^ N-enrichment, these lines of evidence suggest that the Ancistrini may use aggression and territoriality to outcompete non-ancistrin taxa for access to relatively high-quality benthic resources. This, in turn, may have contributed to their taxonomic and functional diversity: The Ancistrini includes almost 30% (243 spp.) of the 831 currently recognized species in Loricariidae [31] and they demonstrate broader jaw morphological diversity than any other tribe in the family [2].

In addition to the paradox of low apparent dietary diversity (Additional file 1: Table S1) accompanied by high jaw diversity [2] that motivated this study, the Loricariidae confront stoichiometric challenges that may partially explain patterns of trophic differentiation that are only chemically discernable. Loricariids have a dense endoskeleton and are covered with dermal plates composed primarily of calcium phosphate (Figure 1), giving them a high physiological demand for dietary phosphorus [10,16]. Paradoxically, the rivers and streams inhabited by loricariids [32] as well as the detritus and biofilm that most loricariids consume [10] tend to be highly P deficient. Discrepancies between dietary P availability and physiological P demand are sufficiently great among the loricariid species investigated to date, that these fishes are the only vertebrates known to have their somatic growth limited by P availability under natural conditions [10]. Given these extreme stoichiometric challenges, loricariid fitness should be particularly sensitive to variation in the C:N:P ratios of food resources [12], and stoichiometric gradients likely provide an important dimension for niche segregation [12,14,15]. Indeed, in the only loricariid assemblage for which stoichiometric data are available, species exhibited interspecific variation along a continuous gradient in whole-body %P [17]. Although relationships between the C:N:P ratios and the isotopic signatures of loricariid trophic resources remain uninvestigated, diversification of aquatic consumers along an δ^15^N/δ^13^C gradient has been shown to be consistent with differential assimilation of dietary components diverging in elemental ratios [15].

The most remarkable and perhaps most stoichiometrically unbalanced dietary specialists within Loricariidae are species of the wood-eating genus *Panaque* (tribe Ancistrini) and the *Hypostomus cochliodon* group (tribe Hypostomini). Both of these distantly related lineages possess specialized jaws and chisel-like teeth (Figure 1B) [1-4], and have gut contents dominated by wood particles [1,4,33]. Nearly parallel isotopic vectors between *Panaque* and the *H. cochliodon* group (Additional file 2: Figure S1) recovered in the analysis of individual vectors suggests that these lineages occupy a similar yet distinctive trophic niche relative to sympatric loricariids. Nearly parallel isotopic vectors between these wood-eaters and the Loricariinae genera *Loricaria* and *Pseudoloricaria*, which are known to consume large fractions of terrestrial seeds, fruits, leaves, and flowers (see Additional file 1: Table S1), supports the correspondence of this isotopically defined niche with a diet of allochthonous plant material. Despite the convergence of wood-eaters on a diet that is rare among vertebrates and unique among fishes, detailed analyses of the digestive physiology of these loricariids has shown them to be unspecialized and functionally similar to non-wood-eating loricariids [33,34]. Loricariids as a whole are largely unable to digest lignocellulose, and instead derive most nutrients and energy from easily digestible breakdown products (e.g., disaccharides and dipeptides) that are produced during microbial degradation of submerged, decomposing wood [1,33,34].

Further examples of correlation between jaw morphology and a lineage’s position in δ^15^N/δ^13^C assemblage isotope space can be seen among the non-wood-eating Ancistrini and Loricariini (Figure 3). Ancistrin genera that are diagnosed by having jaws with fewer, larger, and more centrally clustered teeth (Figure 3A; *Leporacanthicus*, *Lithoxus*, *Pseudacanthicus*, *Scobinancistrus*, *Spectracanthicus*) are more ^15^ N-enriched than genera characterized by having larger numbers of smaller teeth arranged in long rows (Figure 3A; *Ancistrus*, *Baryancistrus*, *Chaetostoma*, *Dekeyseria*, *Lasiancistrus*). These data together with limited gut contents data suggest that the pincer-like jaws of the former group may be specialized for consumption of invertebrates residing in holes and crevices in wood, rocks, and clay nodules in a manner also hypothesized for tube-snouted species of electric fishes [1,35]. In contrast, broad, brush-like jaws of the latter group appear specialized for scraping benthic substrates to dislodge and ingest fractions of detritus and biofilm that are lower in protein content [1]. The Loricariini are distinguished by having jaws that are morphologically and functionally distinct from all other Loricariids [2] and they show a derived preference for relatively ^13^ C-enriched food resources (Figure 3C; Additional file 3: Figure S2) consistent with a dietary preference for the various parts of true plants (i.e., seeds, leaves, flowers; Additional file 1: Table S1). Finally, our conclusions are consistent not only with the isotopic signatures of consumers and with an understanding of jaw functional morphology [2], but also with local-scale variation in the taxonomic and elemental composition of benthic algal turfs, biofilms and detritus [1,10,14,20,36-38].

## Conclusions

Our study introduces the ACSIVA method of visualizing a consumer’s trophic position relative to sympatric taxa in isotope biplot space, and uses this method to integrate isotopic data both spatially across landscapes and evolutionarily across a phylogeny. Our analysis suggests that Loricariidae should be seen not only as a highly diverse phyletic radiation, but also as an ecological radiation that has diversified along trophic niche dimensions that were heretofore cryptic, yet consistent with previously observed jaw morphological diversity [2]. Current understanding of ecological radiation has been heavily influenced by studies of plants and vertebrates that diversified among island archipelagos and lakes, but there are few prominent examples of ecological radiations in river basins or among the detritivores and herbivores that dominate food webs in tropical rivers and virtually all other ecosystems. The frequently amorphous appearance and low taxonomic resolution achievable for gut contents of most herbivores and detritivores may account for our currently poor understanding of niche relationships within this important trophic guild. Detritivores and herbivores appear to select food items based more on chemical and nutritional qualities than taxonomy or morphology [1,10-12,14,15]. By estimating molecular patterns of food resource assimilation over time, stable isotope, fatty acid signature analysis [39], and nutritional physiological approaches [14] provide powerful tools for investigating herbivore and detritivore niche diversification and partitioning.

## Methods

We sampled 32 genera (79 species) of Loricariidae and 1 genus (3 species) of Astroblepidae in 19 assemblages distributed across the Amazon, Orinoco, and Essequibo drainage basins (Figure 4, Table 1; see Additional file 5: Table S2 for a list of species by locality). Each assemblage study site consisted of a reach less than 200 m long, with shallow habitat that was thoroughly sampled on a single day at the end of each region’s dry season by experienced personnel using combinations of nets, rotenone, and electricity. Fish specimens were euthanized by emersion in a 1% solution of tricaine methanesulfonate (MS-222), and then small (<1 gm) samples of postdorsal-fin epaxial muscle were excised and preserved with approximately 2 tsp table salt (NaCl) in small ziplock bags according to standard methods [40]. Specimens from which samples were excised were fixed in 10% formalin and deposited in institutions in North America (Auburn University Museum, Auburn, AL; Academy of Natural Sciences, Philadelphia, PA) and South America (Natural Sciences Museum of Guanare, Venezuela; San Marcos University Natural History Museum, Lima, Peru; University of São Paulo Zoological Museum, Brazil; University of Guyana Biodiversity Center, Georgetown, Guyana). All animal handling was approved by Auburn University Institutional Animal Care and Use Committee protocols 2004–0694 and 2007–1239.

Tissue samples (649 total) were processed following standard protocols [1], with δ^13^C and δ^15^N mass spectrometric isotope analyses performed at the Analytical Chemistry Laboratory, University of Georgia, Athens, using a Carlo Erba CHN elemental analyzer and a Finnigan Delta C mass spectrometer. In order to avoid biases in the calculation of the centroid due to sample-size, all δ^13^C and δ^15^N signatures for each species were grouped and a mean for each species was calculated. These mean species values were then used to compute each local assemblage centroid (See Additional file 4: Figures S3–10 for isotope biplots of all local assemblages examined, including means and standard deviations for all species examined). To determine the statistical significance of the mean vector for a given taxon, all vectors between the assemblage centroid and each individual in an assemblage were calculated trigonometrically, producing 649 individual x assemblage vectors (Figure 2A). For the phylogenetic regressions, genera were used as operational taxonomic units because most represent species with similar trophic ecologies and jaw morphologies [2] and because they represent well-supported clades at the limit of phylogenetic resolution currently available for family Loricariidae. *Hypostomus*, which has a broad range of jaw morphologies, was broken into a group represented by members similar to the type of the genus *H. plecostomus* and the wood-eating species of the *H. cochliodon* group [41].

The 649 vectors were used to run two analyses, an individual-based analysis, and a genus x site analysis: In the individual-based analysis the 649 vectors were subdivided into genera irrespective of site and a single mean vector was calculated for each genus. In the analysis of genus x site means, 120 vectors representing the mean vector for each genus at each site were calculated from the 649 original individual vectors (Figure 2C). In the first analysis, sample sizes were highly unevenly distributed across sites so that results would be biased toward those sites with the highest sample sizes. This was addressed in the second analysis by reducing all individual samples at a given site to a single mean vector. The statistical significance of the mean vector for each genus in each analysis was evaluated using Rayleigh’s Z (Tables 1, 2; Figure 3), and the uniformity of vector distribution was evaluated using Rao’s spacing test (Tables 1, 2). All vector calculations were performed using Oriana software (v4.0 for PC, Kovach Computing Services).

Given the potential that both direction and length of taxon-specific assemblage centroid standardized vectors may vary in response to assemblage size alone, without an accompanying shift in relative trophic position of the taxon, we examined correlations between these variables and assemblage species richness in a limited subset of five genera for which sample sizes allowed statistical tests (Table 3). Tests were conducted on both individual vectors and on genus mean vectors: Individual vectors for three of five genera showed a significant relationship between vector direction and assemblage size, but only one of these (*Chaetostoma*) also had a significant relationship between vector length and assemblage size (Table 3: Test 1). None of these relationships were detected in the analysis of genus mean vectors (Table 3: Test 2). Given that taxon identity is the major independent variable examined herein, and that results of phylogenetic regressions run using both the individual and restricted (genus mean) datasets were almost indistinguishable, systematic effects of assemblage size should be nominal relative to taxon identity and trophic position.

**Table 3:** Test results describing the relationship between assemblage richness and both vector direction (circ. = circular) and vector length (lin. = linear) in a subset of loricariid genera for which sample sizes allowed statistical strength

**Genus**	**Test 1: individual vectors x site**					**Test 2: genus means x site**				
	**n**	**circ.*R2***	**circ.*P***	**lin.*R2***	**lin.*P***	**n**	**circ.*R2***	**circ.*P***	**lin.*R2***	**lin.*P***
*Ancistrus*	44	0.02	0.533	0.05	0.138	9	0.07	0.648	0.05	0.547
*Chaetostoma*	44	0.27	0.000	0.11	0.032	6	0.52	0.174	0.27	0.287
*Hyp. cochliodon* grp	30	0.14	0.022	0.04	0.319	9	0.07	0.669	0.06	0.521
*Hyp. plecostomus* grp	97	0.00	0.671	0.01	0.430	14	0.01	0.872	0.06	0.412
*Spatuloricaria*	17	0.22	0.046	0.00	0.825	6	0.55	0.154	0.02	0.806

Test 1 regressed individual vectors against assemblage richness (n = number of individuals). Test 2 regressed mean genus x site vectors against assemblage richness (n = number of assemblages). Bold face indicates significance at *P* < 0.1.

Ancestral state reconstructions were performed via a phylogenetic least-squares (PLS) criterion [42] that considered both phylogeny [3,43,44] and branch length. Vector data contain both angle (direction) and magnitude (length) components that are not independent; however, vectors can be projected into Cartesian coordinate space yielding X and Y components that are independent. Analyzing these separately preserves both angle and magnitude. Angles were converted to radians in Microsoft Excel (v12.3.2 for Mac OSX). X was calculated as vector length times the cosine of the angle in radians, and Y was calculated as vector length times the sine of the angle in radians. All resulting values for X and Y were between −1 and 1. Given that PLS requires positive values, all values were shifted to positive by adding 1.

A published data matrix [43] was culled in MacClade to include only those genera present in this analysis. In cases where more than one species per genus was present in the phylogenetic data matrix, the type species was retained, if available, or only the first species in the matrix was used. The species in the phylogenetic analysis and this study may not be the same, but there was little intrageneric variation and poor resolution of intrageneric lineage relationships in the phylogenetic analysis [43]. Genus groupings were therefore selected as the primary taxonomic units of comparison in our study. Isotope data were averaged by genus, and to determine if taxon choice factored into the results, the PLS was also run without branch lengths. A tree was built manually in MacClade with identical relationships to the published phylogeny, and branch lengths (number of character changes per branch) were obtained in PAUP* and saved into a tree file; Loricariinae taxa not present in the dataset were added according to relationships in the only published comprehensive phylogeny for Loricariinae [44] and were given a branch length of one because branch lengths in PLS cannot equal zero. X and Y data were added to the phylogenetic matrix in MacClade as continuous characters.

PLS was performed in Mesquite by opening the character matrix and then opening the trees with and without branch lengths. Ancestral values for X and Y were calculated by tracing character history and exporting the resulting values by node. Angles were determined by subtracting one from each value, finding the angle in radians by taking the arctangent of Y/X, converting the angle to degrees (results are from −90° to 90°), and then placing the angle into the correct quadrant by either keeping the same value or adding 180° or 360° as indicated by positive and negative values of X and Y. For visualization on the phylogeny, angular data were segregated into color-coded 45° segments (Figure 2, Additional file 2: Figure S1).

## Authors’ contributions

NKL conceptualized the study and conducted field and laboratory work. NKL, KOW, and JWA wrote the paper. JWA developed and conducted the phylogenetic analyses. All authors read and approved the final manuscript.

## Authors’ information

NKL has conducted research on loricariid biogeography, functional morphology, and trophic ecology for his dissertation and post-doc and is currently working to generate a multi-gene phylogeny for the Ancistrini. KOW has spent 30 years investigating the trophic ecology of tropical river fish communities around the world. JWA completed the first comprehensive, genus-level phylogeny for Loricariidae, and has published extensively on loricariid taxonomy, systematics, and morphology.

## Supplementary Material

Additional file 1 **Table S1.** Summary of published studies of loricariid diets. Click here for file

Additional file 2 **Figure S1.** Mean, individual-based vectors and results of the phylogenetic regression based on these data and color-coded to reflect hypothesized distributions of ancestral lineages along a vertical axis of ^15^ N-enrichment relative to assemblage centroids. Click here for file

Additional file 3 **Figure S2.** Individual- (A) and genus mean- (B) based phylogenetic regressions color-coded to reflect hypothesized distributions of ancestral lineages along a horizontal axis of ^13^ C-enrichment relative to assemblage centroids. Click here for file

Additional file 4 **Figure S3–10.** δ^15^N/δ^13^C isotope biplots of all local assemblages examined. Click here for file

Additional file 5 **Table S2.** List of species by locality. Click here for file
