# Supplementary material for: Trophic diversity in the evolution and community assembly of loricariid catfishes
Source: BMC Evol Biol. 2012 Jul 26;12:124. doi: 10.1186/1471-2148-12-124 (PMC3497581; doi:10.1186/1471-2148-12-124)
Supplement: Additional file 1 — Table S1. Summary of published studies of loricariid diets. [file 1471-2148-12-124-S1.pdf]

Additional File 1: Table 1. Summary of published descriptions of diets of Astroblepidae and Loricariidae. Only dominant constituents are reported where gut contents were reported as extended lists of taxa. Species identity updated to reflect current taxonomy. (nr = not recorded; data from reference 19 used in several subsequent published papers not individually cited here). Ref. = reference.

Species (corrected)	Ref.	Drainage	Country	Method	n	Diet
Astroblepidae						
Astroblepus cyclopus	29	Cauca	Colombia	gut contents	117	36.8% Coleoptera larvae, 15.1% Diptera, 9.9% Trichoptera, 9.9% Odonata
Astroblepus sp.	28	Beni	Bolivia	gut contents	244	100% of stomachs with aquatic insects, 20% algae, 4% sediment, 2% terrestrial insects, 1% plant matter
Hypoptopomatinae						
Hypoptopomatini						
Hypoptopoma sp.	14	Araguaia	Brazil	gut contents	3	detritus
Hypoptopoma sp.	23	Apure	Venezuela	gut contents	52	nr
Hypoptopoma joberti	27	Mamore	Bolivia	gut contents	70	92.9% algae, 5.7% mud, 1.4% insects
Otocinclus cf. *mariae*	20	Napo	Ecuador	gut contents	2	detritus
Otocinclus sp.	23	Apure	Venezuela	gut contents	686	nr
Otothyrini						
Hisonotus sp.	7	Mogi-Guaçu	Brazil	gut contents	128	organic matter, vascular plants, algae
Parotocinclus sp.	14	Araguaia	Brazil	gut contents	117	detritus, other, filamentous algae
Schizolecis guntheri	31	Ribeirão da Serra	Brazil	gut contents	20	sediment, diatoms, macrophyte pieces, algae, Crustacea, Chironomidae, Simuliidae
Hypostominae						
Ancistrini						
Ancistrus chagresi	13	Frijoles	Panama	gut contents	10	detritus
Ancistrus chagresi	19	Frijoles	Panama	behavior	nr	algae
Ancistrus hoplogenys	15	Sinnamary	French Guiana	gut contents	24	vegetative detritus, substratum
Ancistrus hoplogenys	20	Napo	Ecuador	gut contents	4	sand; detritus of undetermined composition

Ancistrus sp.	12	Cinaruco	Venezuela	isotopes	nr	detritus
Ancistrus sp.	23	Apure	Venezuela	gut contents	62	nr
Ancistrus sp.	28	Beni	Bolivia	gut contents	438	94% sediment, 5% algae, 1% seed
Ancistrus sp.	31	Ribeirão da Serra	Brazil	gut contents	14	sediment, diatoms, algae, Chironomidae
Ancistrus triradiatus	11	Apure	Venezuela	gut contents	nr	algae
Chaetostoma dermorhynchum	20	Napo	Ecuador	gut contents	3	detritus
Chaetostoma fischeri	13	Frijoles	Panama	gut contents	6	detritus
Chaetostoma milesi	11	Apure	Venezuela	gut contents	nr	algae
Dekeyseria scaphiryncha	8	Amazon basin	Brazil	isotopes	1	phytoplankton
Dekeyseria scaphiryncha	12	Cinaruco	Venezuela	isotopes	nr	detritus
Hypancistrus inspector	2	Casiquiare	Venezuela	gut contents	~1	detritus, algae, seeds
Lasiancistrus tentaculatus	12	Pasimoni	Venezuela	isotopes	nr	detritus
Lasiancistrus tentaculatus	12	Pasimoni	Venezuela	isotopes	5	algae, detritus, plants
Lithoxus planquettei	26	Sinnamary	French Guiana	gut contents	5	83.3% plant matter, 16.7% Diptera
Megalancistrus aculeatus	6	Parana	Brazil	gut contents	10	60% sponge, 18% organic detritus, 11% sediment, 8% Bryozoa
Panaque albomaculatus	21	Marañon	Peru	gut contents	~1	wood
Panaque cf. *armbrusteri*	17	Tocantins	Brazil	otolith isotopes	22	wood
Panaque cf. *bathyphilus*	30	Marañon	Peru	gut contents	11	75% wood, 17% amorphous detritus, 6% diatoms, 1% algae/sediment
Panaque dentex	21	Marañon	Peru	gut contents	~1	wood
Panaque gnomus	21	Marañon	Peru	gut contents	~1	wood
Panaque maccus	21	Apure	Venezuela	gut contents	~1	wood
Panaque nigrolineatus	20	Napo	Ecuador	gut contents	1	plant debris
Panaque nigrolineatus	21	Apure	Venezuela	gut contents	~1	wood
Panaque nocturnus	30	Marañon	Peru	gut contents	11	70% wood, 18% amorphous detritus, 8% diatoms, 2% algae, 2% sediment
Panaque nocturnus	21	Marañon	Peru	gut contents	~1	wood
Panaque purusiensis	21	Purus	Peru/Brazil	gut contents	~1	wood

Pseudancistrus niger	26	Sinnamary	French Guiana	gut contents	47	55.9% mud, 23.3% chitin, 17.6% diptera, 11.8% sand, 2.9% Arachnidae
Hypostomini						
Hemiancistrus aspidolepis	13	Frijoles	Panama	gut contents	2	detritus
Hemiancistrus aspidolepis	19	Frijoles	Panama	behavior	nr	algae
Hemiancistrus aspidolepis	25	Pedro Miguel	Panama	gut contents	1	algae
Hypostomus ancistroides	7	Mogi-Guaçu	Brazil	gut contents	13	organic matter, vascular plants, algae
Hypostomus argus	23	Apure	Venezuela	gut contents	379	algae, detritus, microscopic animals
Hypostomus cochliodon	3	Paraguay	Brazil/Paraguay	gut contents	~1	wood
Hypostomus commersoni	35	La Plata	Argentina	gut contents	nr	inorganic detritus, diatoms, organic detritus
Hypostomus emarginatus	14	Araguaia	Brazil	gut contents	3	detritus
Hypostomus emarginatus	33	Tocantins	Brazil	gut contents	60	detritus, unicellular algae, sediment
Hypostomus ericae	14	Araguaia	Brazil	gut contents	2	other (wood?)
Hypostomus ericius	3	Marañon	Peru	gut contents	~1	wood
Hypostomus garmani	32	São Francisco	Brazil	gut contents	20	50% organic matter, 50% algae
Hypostomus gymnorhynchus	16	Mahury	French Guiana	gut contents	14	100% detritus
Hypostomus hemicochliodon	3	Amazon basin	Peru/Ecuador/Brazil	gut contents	~1	wood, detritus
Hypostomus hondae	3	Maracaibo/Magdalena	Colombia/Venezuela	gut contents	~1	wood
Hypostomus levis	3	Beni	Bolivia	gut contents	~1	wood
Hypostomus levis	28	Beni	Bolivia	gut contents	39	100% sediment, 18% algae
Hypostomus margaritifer	6	Parana	Brazil	gut contents	10	49% Bryophyta, 21% organic detritus, 18% sediment, 10% Rhodophyta
Hypostomus microstomus	6	Parana	Brazil	gut contents	10	60% sponge, 16% sediment, 11% organic detritus, 3% plant detritus
Hypostomus oculeus	3	Upper Amazon	Peru/Ecuador	gut contents	~1	wood
Hypostomus pagei	3	Aroa/Yaracuy	Venezuela	gut contents	~1	wood
Hypostomus plecostomoides	3	Orinoco	Colombia/Venezuela	gut contents	~1	wood
Hypostomus plecostomoides	23	Apure	Venezuela	gut contents	6	nr
Hypostomus plecostomus	8	Amazon basin	Brazil	isotopes	1	detritus

Hypostomus plecostomus	10	Orinoco	Venezuela	isotopes	1-10	herbivore
Hypostomus plecostomus	20	Napo	Ecuador	gut contents	5	detritus, sphaerid clams
Hypostomus plecostomus	26	Sinnamary	French Guiana	gut contents	3	50% mud, 50% sand
Hypostomus pyrineusi	3	Upper Amazon	Peru/Ecuador/Brazil	gut contents	~1	wood
Hypostomus regani	6	Parana	Brazil	gut contents	10	71% detritus, 21% sediment, 7% plant detritus
Hypostomus regani	17	Mogi-Guaçu	Brazil	otolith isotopes	14	algae
Hypostomus sculpodon	3	Negro/Orinoco	Venezuela/Brazil	gut contents	~1	wood, detritus
Hypostomus sp.	28	Beni	Bolivia	gut contents	96	91% sediment, 9% algae, 1% plant
Hypostomus sp. a1	1	São Francisco	Brazil	gut contents	nr	detritus
Hypostomus sp. a2	1	São Francisco	Brazil	gut contents	nr	detritus
Hypostomus sp. b1	14	Araguaia	Brazil	gut contents	33	detritus, filamentous algae, other
Hypostomus sp. b2	14	Araguaia	Brazil	gut contents	6	detritus
Hypostomus sp. b3	14	Araguaia	Brazil	gut contents	17	detritus
Hypostomus sp. b4	14	Araguaia	Brazil	gut contents	12	detritus
Hypostomus spp. (seven)	22	Parana	Brazil	guts & isotopes	nr	detritus
Hypostomus strigaticeps	5	Curumbataí	Brazil	gut contents	938	diatoms, fungal hyphae, chlorophytes, cyanophytes
Hypostomus taphorni	3	Essequibo	Venezuela/Guyana	gut contents	~1	wood
Hypostomus ternetzi	6	Parana	Brazil	gut contents	10	42% Bryozoa, 32% sediment, 23% organic detritus
Pterygoplichthini						
Pterygoplichthys sp.	27	Mamore	Bolivia	gut contents	8	50% mud, 25% algae, 12.5% plant
Pterygoplichthys disjunctivus	30	Wekiva Springs	Florida, USA	gut contents	17	40% detritus, 23% diatoms, 22% algae, 10% sediment
Pterygoplichthys multiradiatus	23	Apure	Venezuela	gut contents	479	nr
Pterygoplichthys pardalis	24	Amazon basin	Brazil	gut contents	29	detritus
Pterygoplichthys radiatus	8	Amazon basin	Brazil	isotopes	5	detritus
Rhinelepini						
Rhinelepis aspera	6	Parana	Brazil	gut contents	10	96% detritus, 4% sediment

Loricariinae						
Farlowellini						
Farlowella kneri	20	Napo	Ecuador	gut contents	2	detritus
Farlowella sp.	14	Araguaia	Brazil	gut contents	20	detritus, filamentous algae, insects
Farlowella sp.	23	Apure	Venezuela	gut contents	1	nr
Harttiini						
Harttia kronei	31	Ribeirão da Serra	Brazil	gut contents	20	sediment, diatoms, algae, macrophyte pieces, Crustacea, Chironomidae
Harttia sp.	32	São Francisco	Brazil	gut contents	22	50% organic matter, 45% algae, 5% aquatic insects
Harttia surinamensis	26	Sinnamary	French Guiana	gut contents	23	58.1% mud, 19.5% chitinous remains, 19.4% sand
Sturisoma nigrirostrum	14	Araguaia	Brazil	gut contents	8	detritus, other, leaves and flowers
Sturisoma nigrirostrum	27	Mamore	Bolivia	gut contents	52	100% algae, 9.6% mud
Sturisoma sp.	23	Apure	Venezuela	gut contents	1	nr
Loricariini						
Crossoloricaria sp.	4	not cited	not cited	gut contents	~1	seeds
Loricaria cataphracta	16	Mahury	French Guiana	gut contents	21	45.7% aquatic inverts., 28.1% detritus, 21% higher plants, 13.3% terr. inverts., 12.9% plankton
Loricaria cataphracta	20	Napo	Ecuador	gut contents	1	detritus
Loricaria filamentosa	20	Napo	Ecuador	gut contents	3	detritus
Loricaria sp.	4	not cited	not cited	gut contents	~1	seeds
Loricaria sp.	14	Araguaia	Brazil	gut contents	35	fruits and seeds, detritus, other, leaves and flowers, aquatic insects
Loricariichthys anus	35	La Plata	Argentina	gut contents	nr	inorganic and organic detritus, diatoms, Chironomidae, filamentous algae, molluscs, crustaceans
Loricariichthys platymetopon	8	Amazon basin	Brazil	isotopes	3	nr
Loricariichthys platymetopon	9	Parana	Brazil	gut contents	116	organic detritus, chironomids

Loricariichthys platymetopon	18	Parana	Brazil	gut contents	49	detritus, sediment, organic detritus, diatoms
Loricariichthys typus	23	Apure	Venezuela	gut contents	501	large fractions of plant and animal material
Paraloricaria vetula	35	La Plata	Argentina	gut contents	nr	molluscs, inorganic detritus, diatoms, crustaceans, filamentous algae, Chironomidae
Pseudohemiodon cf. *laticeps*	20	Napo	Ecuador	gut contents	2	insect debris, caddisfly larvae, snail
Pseudohemiodon laticeps	27	Mamore	Bolivia	gut contents	20	100% algae, 70% mud
Pseudoloricaria sp.	14	Araguaia	Brazil	gut contents	47	filamentous algae, detritus, aquatic insects, leaves and flowers
Rineloricara lanceolata	20	Napo	Ecuador	gut contents	2	aquatic plants, detritus
Rineloricaria caracasensis	12	Aguaro, Apure	Venezuela	isotopes	nr	detritus
Rineloricaria caracasensis	12	Aguaro, Apure	Venezuela	isotopes	4	algae, detritus, plants
Rineloricaria caracasensis	12	Apure	Venezuela	isotopes	5	algae, detritus, plants
Rineloricaria caracasensis	23	Apure	Venezuela	gut contents	628	nr
Rineloricaria uracantha	13	Frijoles	Panama	gut contents	8	detritus, aquatic invertebrates
Rineloricaria uracantha	19	Frijoles	Panama	behavior	nr	algae
Spatuloricaria caquetae	20	Napo	Ecuador	gut contents	1	detritus
Spatuloricaria evansi	34	Maranhão	Brazil	gut contents	5	sediments, diatoms, Coleoptera, Trichoptera, Ephemeroptera, Simuliidae, Lepidoptera
Spatuloricaria sp.	14	Araguaia	Brazil	gut contents	12	fruits and seeds, aquatic insects, detritus, arthropod, terrestrial insects
Neoplecostominae						
Kronichthys heylandi	31	Ribeirão da Serra	Brazil	gut contents	30	sediment, diatoms, algae, macrophyte pieces, Crustacea, Chironomidae

Literature Cited:

1. Alvim MC, Peret AC: **Food resources sustaining the fish fauna in a section of the upper São Francisco River in Três Marias, MG, Brazil.** *Brazilian Journal of Biology* 2004, **64**:195-202.

2. Armbruster JW: ***Hypancistrus inspector*, a new species of suckermouth armored catfish (Loricariidae: Ancistrinae).** *Copeia* 2002, **2002**:86-92.
3. Armbruster JW: **The species of the *Hypostomus cochliodon* group (Siluriformes: Loricariidae).** *Zootaxa* 2003, **249**:1-60.
4. Armbruster JW: **Phylogenetic relationships of the suckermouth armoured catfishes (Loricariidae) with emphasis on the Hypostominae and the Ancistrinae.** *Zoological Journal of the Linnean Society* 2004, **141**:1-80.
5. Cardone IB, Lima-Junior SE, Goitein R: **Diet and capture of *Hypostomus strigaticeps* (Siluriformes, Loricariidae) in a small Brazilian stream: relationship with limnological aspects.** *Brazilian Journal of Biology* 2006, **66**:25-33.
6. Delariva RL, Agostinho AA: **Relationships between morphology and diets of six neotropical loricariids.** *Journal of Fish Biology* 2001, **58**:832-847.
7. Ferreira KM: **Biology and ecomorphology of stream fishes from the rio Mogi-Guaçu basin, Southeastern Brazil.** *Neotropical Ichthyology* 2007 **5**:311-326.
8. Forsberg BR, Araujo-Lima CARM, Martinelli LA, Victoria RL, Bonassi JA: **Autotrophic carbon sources for fish of the Central Amazon.** *Ecology* 1993, **74**:643-652.
9. Fugi R, Agostinho AA, Hahn NS: **Trophic morphology of five benthic-feeding fish species of a tropical floodplain.** *Revista Brasileira de Biologia* 2001, **61**:27-33.
10. Hamilton SK, Sippel SJ, Bunn SE: **Separation of algae from detritus for stable isotope or ecological stoichiometry studies using density fractionation in colloidal silica.** *Limnology and Oceanography: Methods* 2005, **3**:149-157.
11. Hood JM, Vanni MJ, Flecker AS: **Nutrient recycling by two phosphorus rich grazing catfish: the potential for phosphorus-limitation of fish growth.** *Oecologia* 2005, **146**:247-257.
12. Jepsen DB, Winemiller KO: **Structure of tropical river food webs revealed by stable isotope ratios.** *Oikos* 2002, **96**:46-55.
13. Kramer DL, Bryant MJ: **Intestine length in the fishes of a tropical stream: 1. Ontogenetic allometry.** *Environmental Biology of Fishes* 1995, **42**:115-127.
14. Melo CE, Arruda Machado F, Pinto-Silva V: **Feeding habits of fish from a stream in the savanna of Central Brazil, Araguaia Basin.** *Neotropical Ichthyology* 2004, **2**:37-44.
15. Mérigoux S, Ponton D, Mérona B: **Fish richness and species-habitat relationships in two coastal streams of French Guiana, South America.** *Environmental Biology of Fishes* 1998, **51**:25-39.
16. Mérona B, Hugueny B, Tejerina-Garro FL: **Diet-morphology relationship in a fish assemblage from a medium-sized river of French Guiana: the effect of species taxonomic proximity.** *Aquatic Living Resources* 2008, **21**:171-184.
17. Nonogaki H, Nelson JA, Patterson WP: **Dietary histories of herbivorous loricariid catfishes: evidence from $\delta^{13}C$ values of otoliths.** *Environmental Biology of Fishes* 2007, **78**:13-21.
18. Peretti D, Fatima Andrian I: **Trophic structure of fish assemblages in five permanent lagoons of the high Paraná River floodplain, Brazil.** *Environmental Biology of Fishes* 2004, **71**:95-103.
19. Power ME: **The grazing ecology of armored catfish (Loricariidae) in a Panamanian stream.** PhD dissertation 1981, University of Washington, Seattle.
20. Saul WG: **An ecological study of fishes at a site in upper Amazonian Ecuador.** *Proceedings of the Academy of Natural Sciences of Philadelphia* 1975, **127**:93-134.
21. Schaefer SA, Stewart DJ: **Systematics of the *Panaque dentex* species group (Siluriformes: Loricariidae), wood-eating armored catfishes**

from tropical South America. *Ichthyological Exploration of Freshwaters* 1993, **4**:309-342.
22. Vaz MM, Petrere Jr. M, Martinelli LA, Mozeto AA: **The dietary regime of detritivorous fish from the river Jacaré Pepira, Brazil.** *Fisheries Management and Ecology* 1999, **6**:121-132.
23. Winemiller KO: **Spatial and temporal variation in tropical fish trophic networks.** *Ecological Monographs* 1990, **60**:331-367.
24. Yossa MI, Araujo-Lima CA. **Detritivory in two Amazonian fish species.** *Journal of Fish Biology* 1998, **52**:1141-1153.
25. Zaret TM, Rand AS: **Competition in tropical stream fishes: support for the competitive exclusion principle.** *Ecology* 1971, **52**:336-342.
26. Horeau V, Cerdan P, Champeau A, Richard]S: **Importance of aquatic invertebrates in the diet of rapids-dwelling fish in the Sinnamary River, French Guiana.** *Journal of Tropical Ecology* 1998, **14**:851-864.
27. Pouilly M, Yunoki T, Rosales C, Torres L: **Trophic structure of fish assemblages from Mamoré River floodplain lakes (Bolivia).** *Ecology of Freshwater Fish* 2004, **13**: 245-257.
28. Pouilly M, Barrera S, Rosales C: **Changes of taxonomic and trophic structure of fish assemblages along an environmental gradient in the Upper Beni watershed (Bolivia).** *Journal of Fish Biology* 2006, **68**:137-156.
29. Román-Valencia C: **Trophic and reproductive ecology of *Trichomycterus caliense* and *Astroblepus cyclopus* (Pisces: Siluriformes) in the Quidio River, Upper Cauca, Colombia.** *Revista de Biología Tropical* 2001, **49**:657-666.
30. German DP: **Inside the guts of wood-eating catfishes: can they digest wood?** *Journal of Comparative Physiology B* 2009, **179**:1011-1023.
31. Buck S, Sazima I: **An assemblage of mailed catfishes (Loricariidae) in southeastern Brazil: distribution, activity, and feeding.** *Ichthyological Exploration of Freshwaters* 1995, **6**:325-332.
32. Casatti L, Castro RMC: **A fish community of the São Francisco River headwaters riffles, southeastern Brazil.** *Ichthyological Exploration of Freshwaters* 1998, **9**:229-242.
33. Castro ALM, Abrecht MP, Pellegrini-Caramaschi É: **Diet of *Hypostomus emarginatus* (Teleostei; Loricariidae) in the upper Tocantins river before and after impoundment by the Serra da Mesa Hydroelectric Dam.** *Biociências, Porto Alegre* 2003, **11**:23-30.
34. Rapp Py-Daniel L, Py-Daniel V: **Observações sobre *Spatuloricaria evansi* (Boulenger, 1892) (Osteichthys; Loricariidae) e a sua predação em Simuliidae (Diptera; Culicomorpha).** *Boletim do Museu Paraense Emilio Goeldi Zoologia* 1984, **1**:207-218.
35. Angelescu V, Gneri FS: **Adaptaciones del aparato digestivo al régimen alimenticio en algunos peces del río Uruguay y del río de la Plata.** *Revista del Instituto Nacional de Investigacion de las Ciencias Naturales* 1949, **1**:161-272.
