# Supplementary figures and images for: Trophic diversity in the evolution and community assembly of loricariid catfishes
Source: BMC Evol Biol. 2012 Jul 26;12:124. doi: 10.1186/1471-2148-12-124 (PMC3497581; doi:10.1186/1471-2148-12-124)

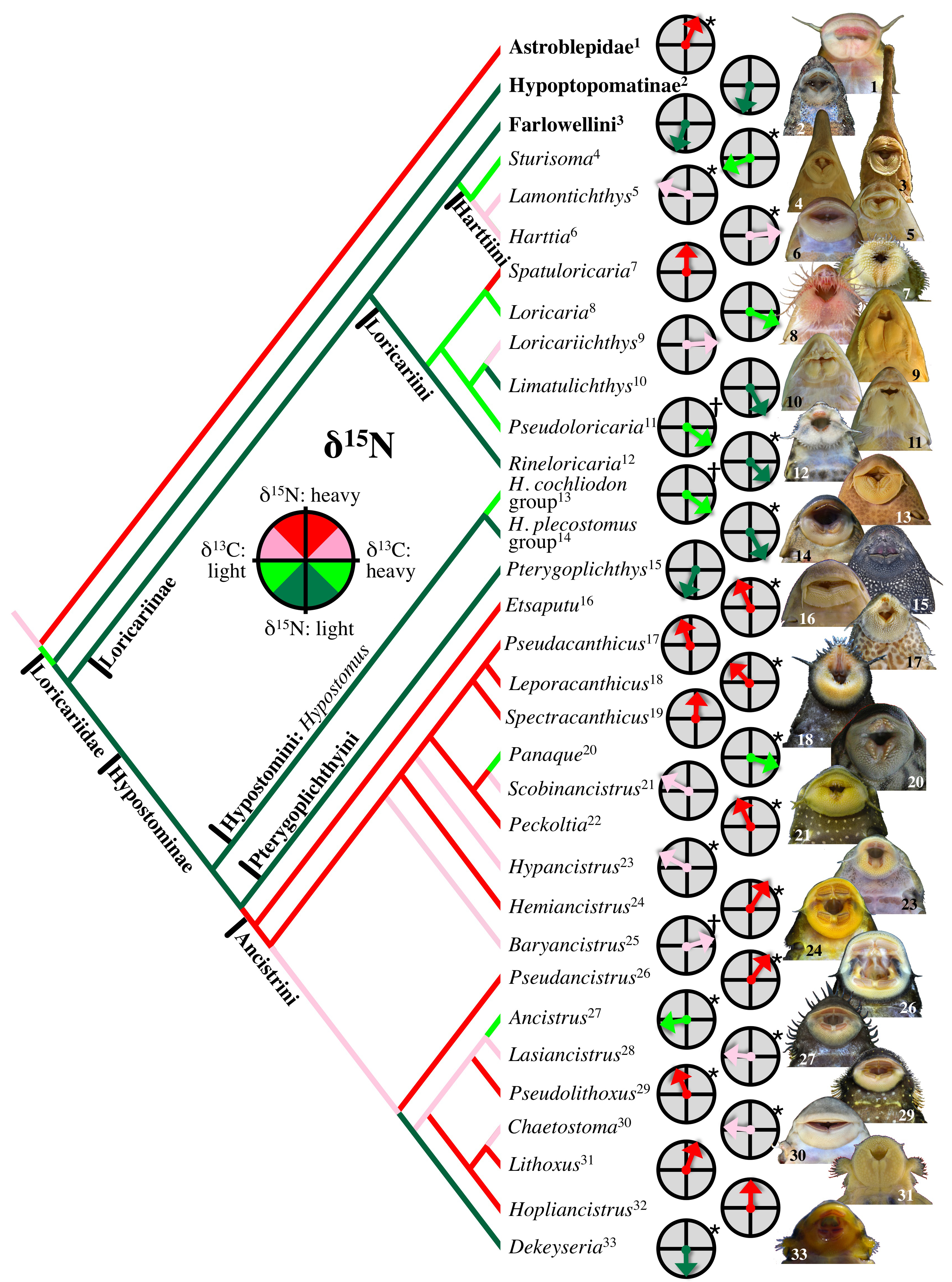

Supplement: Additional file 2 — Figure S1. Mean, individual-based vectors and results of the phylogenetic regression based on these data and color-coded to reflect hypothesized distributions of ancestral lineages along a vertical axis of 15 N-enrichment relative to assemblage centroids. [file 1471-2148-12-124-S2.jpeg]

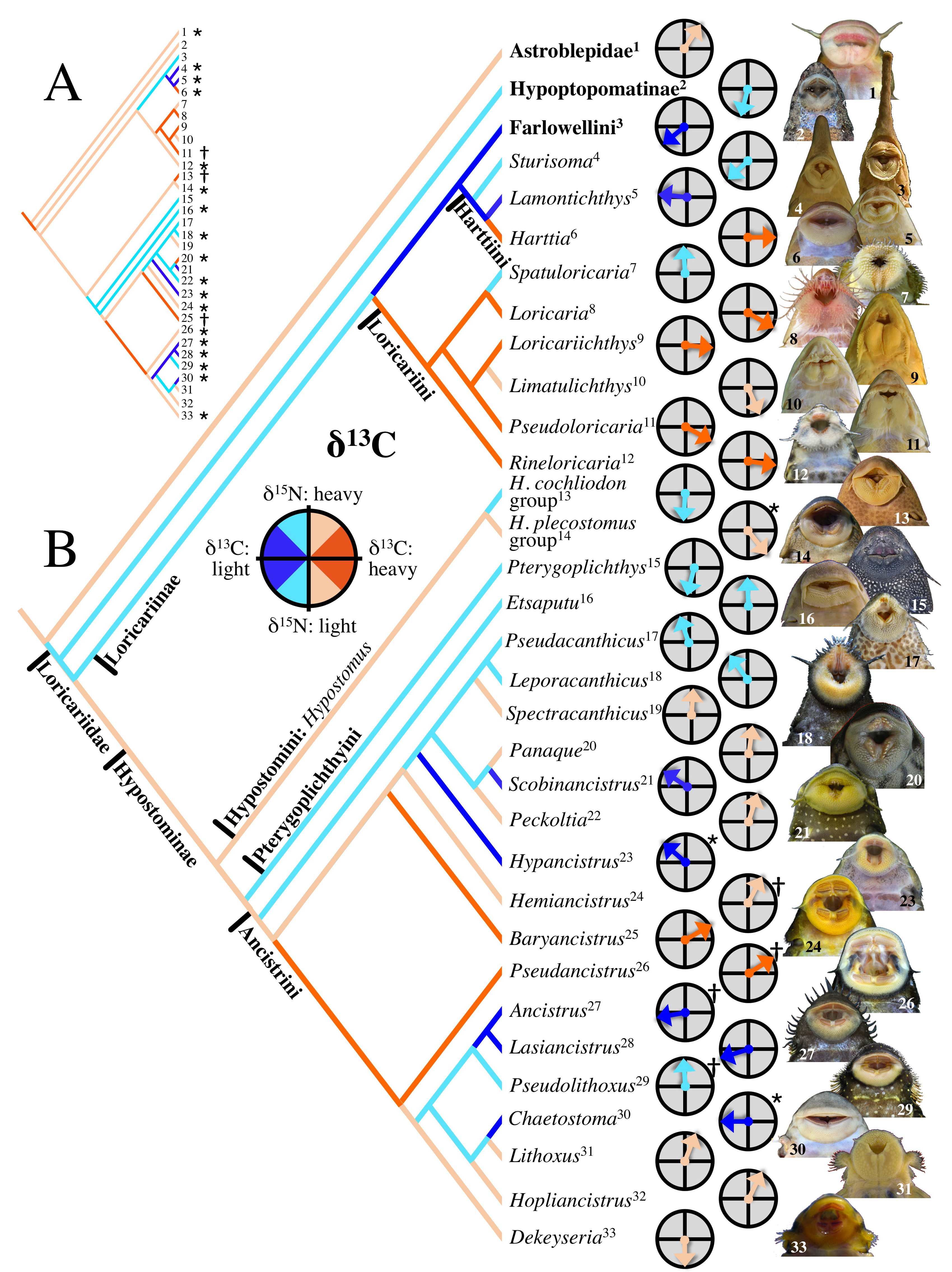

Supplement: Additional file 3 — Figure S2. Individual- (A) and genus mean- (B) based phylogenetic regressions color-coded to reflect hypothesized distributions of ancestral lineages along a horizontal axis of 13 C-enrichment relative to assemblage centroids. [file 1471-2148-12-124-S3.jpeg]
