# Supplementary material for: Trophic diversity in the evolution and community assembly of loricariid catfishes
Source: BMC Evol Biol. 2012 Jul 26;12:124. doi: 10.1186/1471-2148-12-124 (PMC3497581; doi:10.1186/1471-2148-12-124)
Supplement: Additional file 4 — Figure S3–10. δ15N/δ13C isotope biplots of all local assemblages examined. [file 1471-2148-12-124-S4.pdf]

Additional file 3. Table 2 Taxonomic and geographic distribution of species sampled in this study.

				Brazil			Guyana				Peru								Venezuela			
**Family**	**Subfamily**	**Tribe**	**Species**	**Peixoto**	**Tapajos**	**Xingu**	**Bununi**	**Essequibo**	**Rupununi**	**Takutu**	**Almendro**	**Huancabamba**	**Marañon**	**Nieva**	**Shaapan**	**Siasme (lower)**	**Siasme (upper)**	**Utcubamba**	**Casiquiare**	**Orinoco-1**	**Orinoco-2**	**Ventuari**
Astroblepidae																						
			Astroblepus sp. 1								•											
			Astroblepus sp. 2									•										
			Astroblepus sp. 3														•					
Loricariidae																						
	Hypoptopomatinae																					
		Hypoptopomatini																				
			Hypoptopoma guianense				•	•														
	Hypostominae																					
		Ancistrini																				
			Ancistrus macrophthalmus																	•	•	
			Ancistrus sp.			•																
			Ancistrus sp. 'longjaw'								•											
			Ancistrus sp. 'plain'																		•	•
			Ancistrus sp. 'shortjaw'													•						
			Ancistrus sp. 'wormline'									•										
			Ancistrus temminckii				•			•												
			Baryancistrus beggini																	•		•
			Baryancistrus demantoides																	•		
			Baryancistrus sp. "B&W"		•																	
			Baryancistrus sp. "gold nugget"			•																
			Chaetostoma lineopunctatum														•					
			Chaetostoma microps								•		•				•	•				
			Chaetostoma sp. 1									•										
			Chaetostoma sp. 2															•				
			Dekeyseria scaphirhyncha																			•
			Etsaputu relictum										•	•								
			Hemiancistrus guahiborum																•		•	•
			Hemiancistrus sabaji																		•	
			Hemiancistrus sp. "gold spot"			•																
			Hemiancistrus subviridis																•	•	•	•
			Hopliancistrus tricornis		•																	
			Hypancistrus contradens																	•		•
			Hypancistrus furunculus																	•		•
			Hypancistrus inspector																•			
			Hypancistrus lunaorum																		•	
			Lasiancistrus schomburgkii										•	•				•				
			Lasiancistrus tentaculatus																			•
			Leporacanthicus galaxias																	•		•
			Leporacanthicus triactis																			•
			Lithoxus lithoides					•														
			Panaque albomaculatus										•									
			Panaque armbrusteri	•	•	•																
			Panaque cf. *bathyphilus*										•									
			Panaque cf. *maccus*							•												
			Panaque gnomus										•									
			Panaque nocturnus										•									
			Peckoltia braueri							•												
			Peckoltia cavatica						•													
			Peckoltia snethlegeae		•																	
			Peckoltia sp. 'big spot'			•																
			Peckoltia vermiculata																			•
			Pseudacanthicus leopardus						•													
			Pseudancistrus nigrescens					•														

Pseudancistrus pectegenitor																•	•		
Pseudancistrus sidereus																•		•	
Pseudolithoxus anthrax																	•		•
Pseudolithoxus dumus																		•	
Pseudolithoxus tigris																	•		
Scobinancistrus sp.		•																	
Spectracanthicus puntatissimus			•																
Hypostomini																			
Hypostomus (Cochliodon) macushi						•													
Hypostomus (Cochliodon) pyrineusi										•			•		•				
Hypostomus (Cochliodon) sp. "dirty"	•	•	•																
Hypostomus (Cochliodon) sp. "spots"			•																
Hypostomus (Cochliodon) taphorni																			
Hypostomus cf. *emarginatus*	•	•																	
Hypostomus emarginatus											•								
Hypostomus hemiurus					•														
Hypostomus niceforoi								•		•			•						
Hypostomus rhantos																	•	•	•
Hypostomus sp.		•	•																
Hypostomus squalinus						•	•									•		•	
Hypostomus unicolor										•	•								
Pterygoplichthyini																			
Pterygoplichthys gibbiceps																•		•	
Loricariinae																			
Farlowellini																			
Farlowella acus				•															
Farlowella amazona										•	•								
Harttiini																			
Harttia platystoma					•														
Harttia sp.			•																
Lamontichthys filamentosus										•									
Sturisoma monopelte						•													
Sturisoma nigrirostrum										•									
Loricariini																			
Limatulichthys griseus					•	•	•												
Loricaria clavipinna										•	•								
Loricaria sp. 1						•													
Loricaria sp. 2		•	•																
Loricariichthys brunneus						•													•
Pseudoloricaria sp.		•																	
Rineloricaria fallax				•		•													
Rineloricaria lanceolata								•		•		•							
Rineloricaria sp.						•													
Rineloricaria stewarti						•													
Spatuloricaria puganensis										•		•			•				
Spatuloricaria sp. 1					•														
Spatuloricaria sp. 2		•	•																
