# Supplementary material for: Trophic diversity in the evolution and community assembly of loricariid catfishes
Source: BMC Evol Biol. 2012 Jul 26;12:124. doi: 10.1186/1471-2148-12-124 (PMC3497581; doi:10.1186/1471-2148-12-124)

Additional File 5

Figure 3 δ^{15}N and δ^{13}C relationships of loricariid plus astroblepid consumers in (A) Shaapan creek, Marañon (Amazon) drainage, northern Peru; (B) Peixoto river, Tapajós (Amazon) drainage, Brazil; (C) upper Siasme creek, Marañon (Amazon) drainage, northern Peru; and (D) Bununi creek, Rupununi (Essequibo) drainage, Guyana. Sample sizes in parentheses.

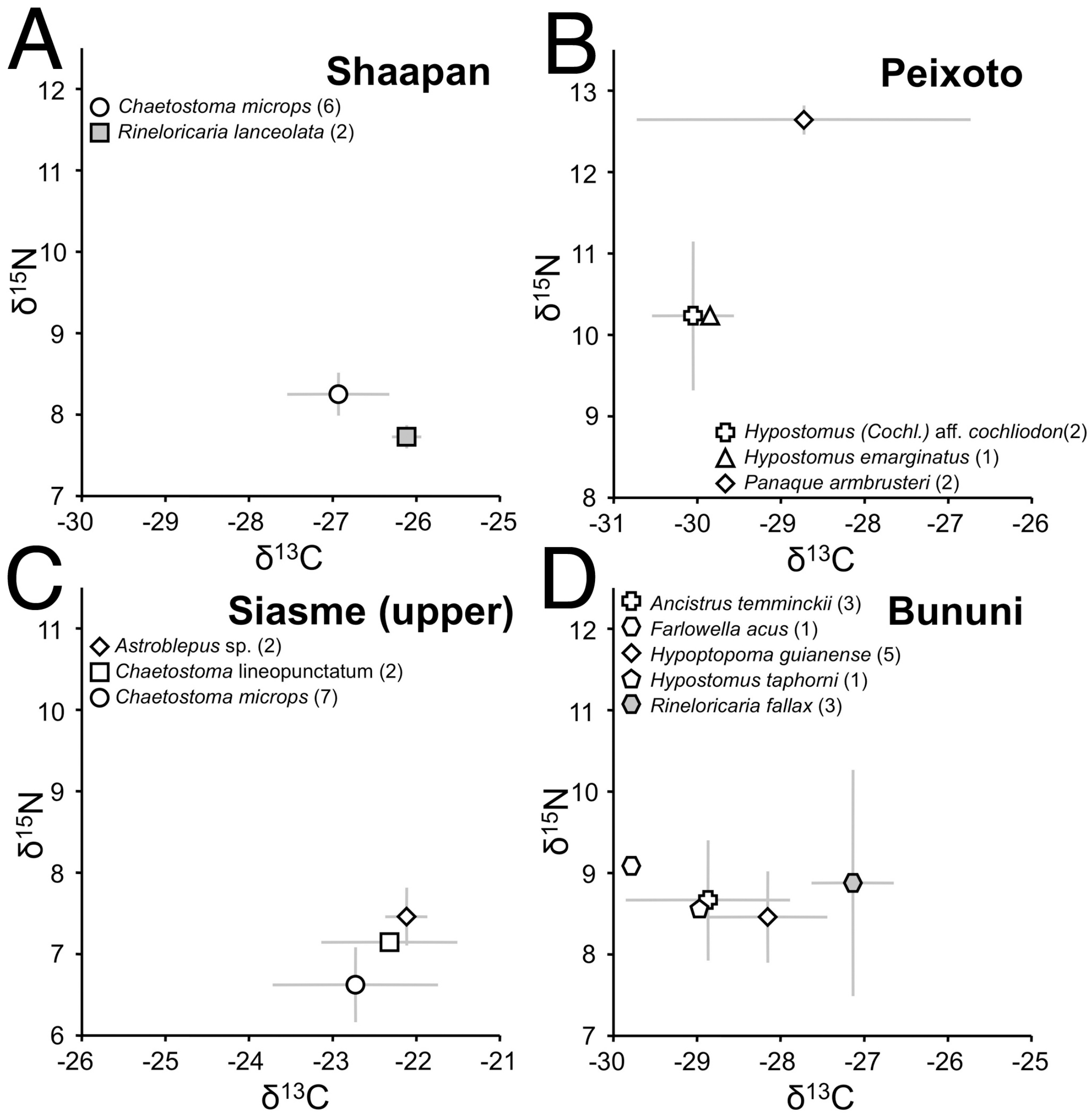

Figure 4 $\delta^{15}N$ and $\delta^{13}C$ relationships of loricariid consumers in (A) the Utcubamba river, Marañon (Amazon) drainage, northern Peru; (B) Casiquiare canal, Negro (Amazon) drainage, southern Venezuela; and (C) Takutu river, Branco (Amazon) drainage, Guyana. Sample sizes in parentheses.

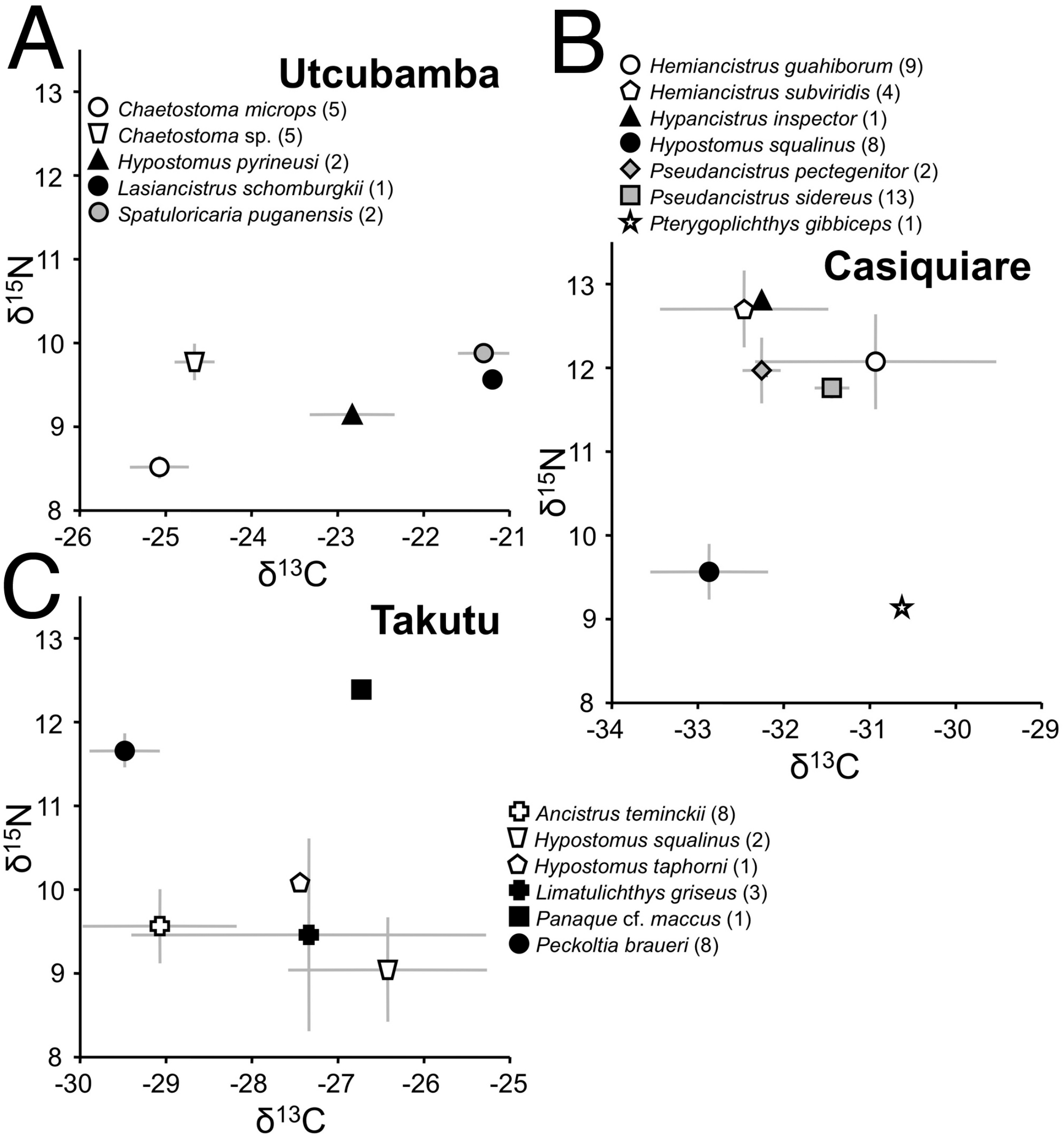

Figure 5 δ^{15}N and δ^{13}C relationships of loricariid plus astroblepid consumers in (A) the Huancabamba river, and (B) lower Siasme creek, both Marañon (Amazon) drainages in northern Peru. Sample sizes in parentheses.

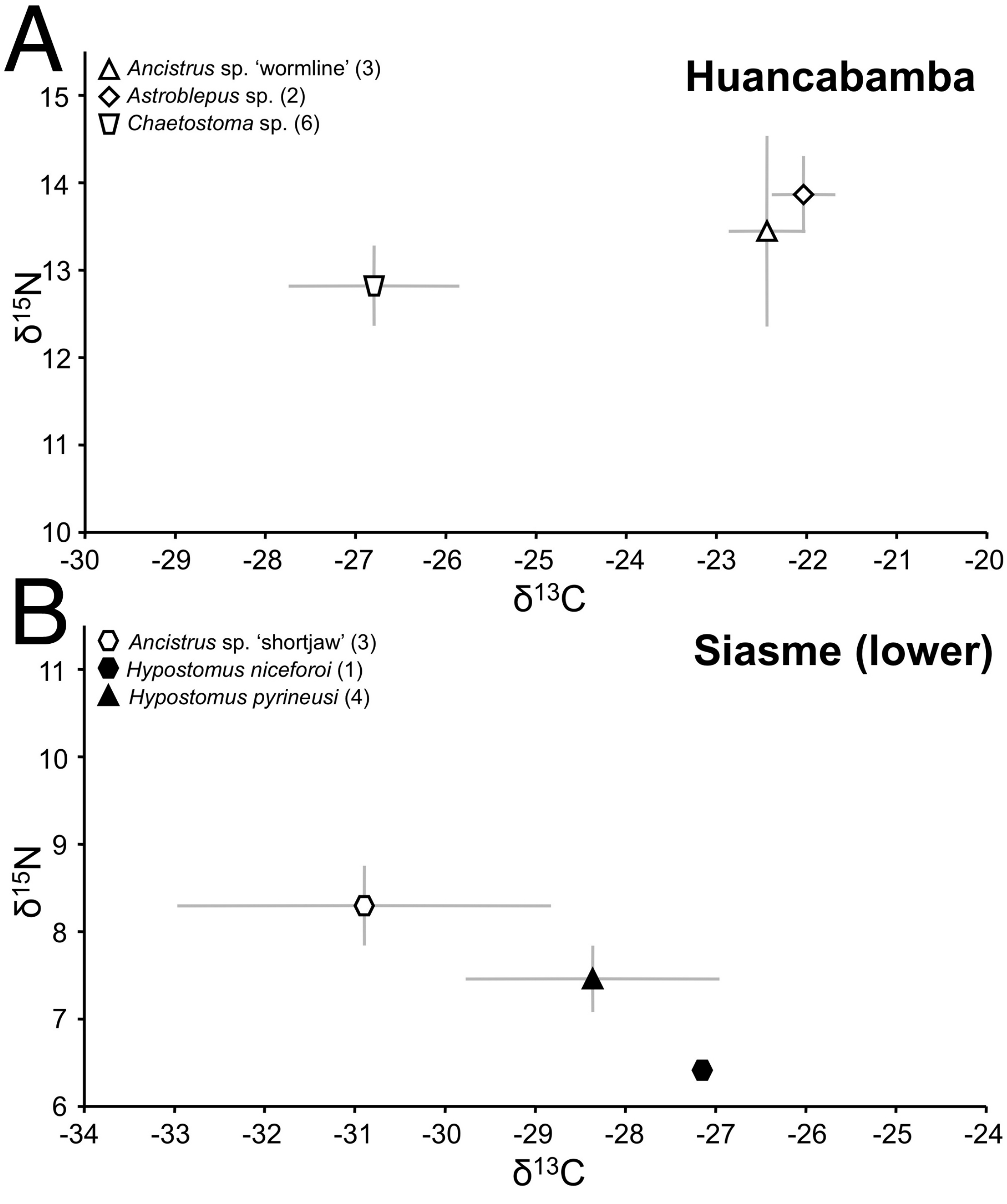

Figure 6 δ^{15}N and δ^{13}C relationships of loricariid plus astroblepid consumers in (A) Almendro creek, and (B) the Nieva river, both Marañon (Amazon) drainages in northern Peru. Sample sizes in parentheses.

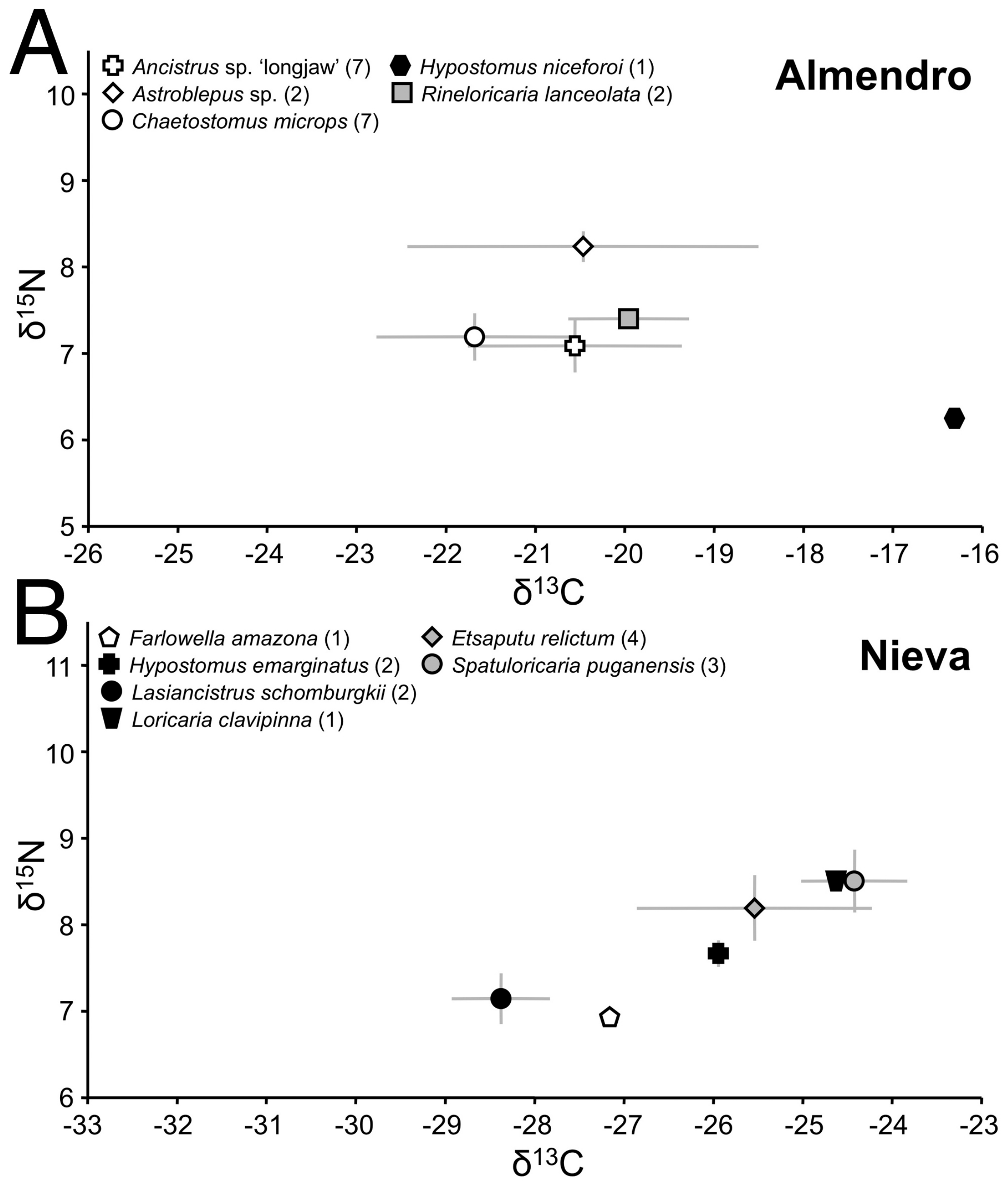

Figure 7 $\delta^{15}N$ and $\delta^{13}C$ relationships of loricariid consumers in the (A) Essequibo river, Guyana, and (B) the Jamanxim river, Tapajos (Amazon) drainage, Brazil. Sample sizes in parentheses.

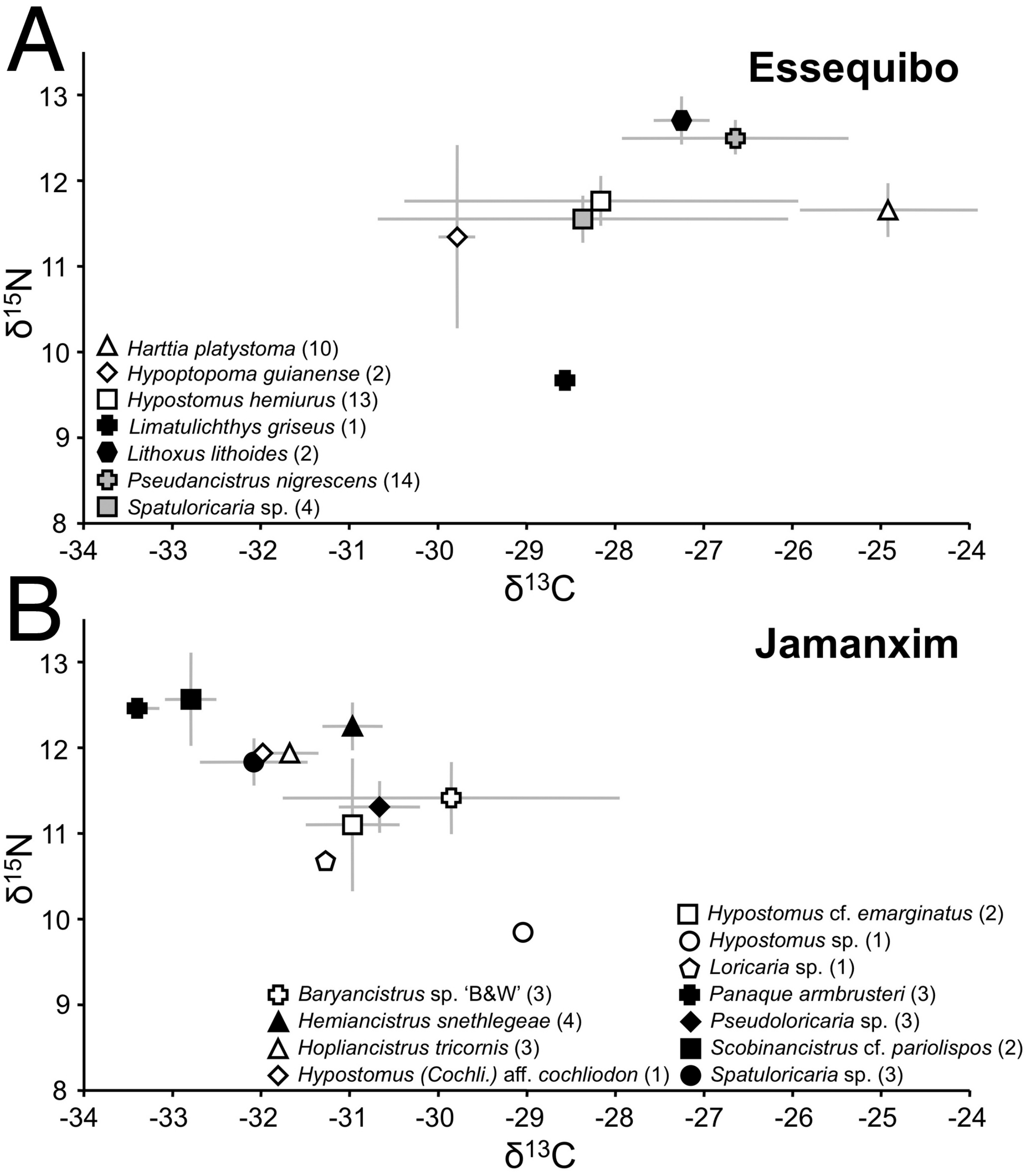

Figure 8 δ^{15}N and δ^{13}C relationships of loricariid consumers in the Orinoco river main channel, (A) below and (B) above the mouth of the Ventuari river. Sample sizes in parentheses.

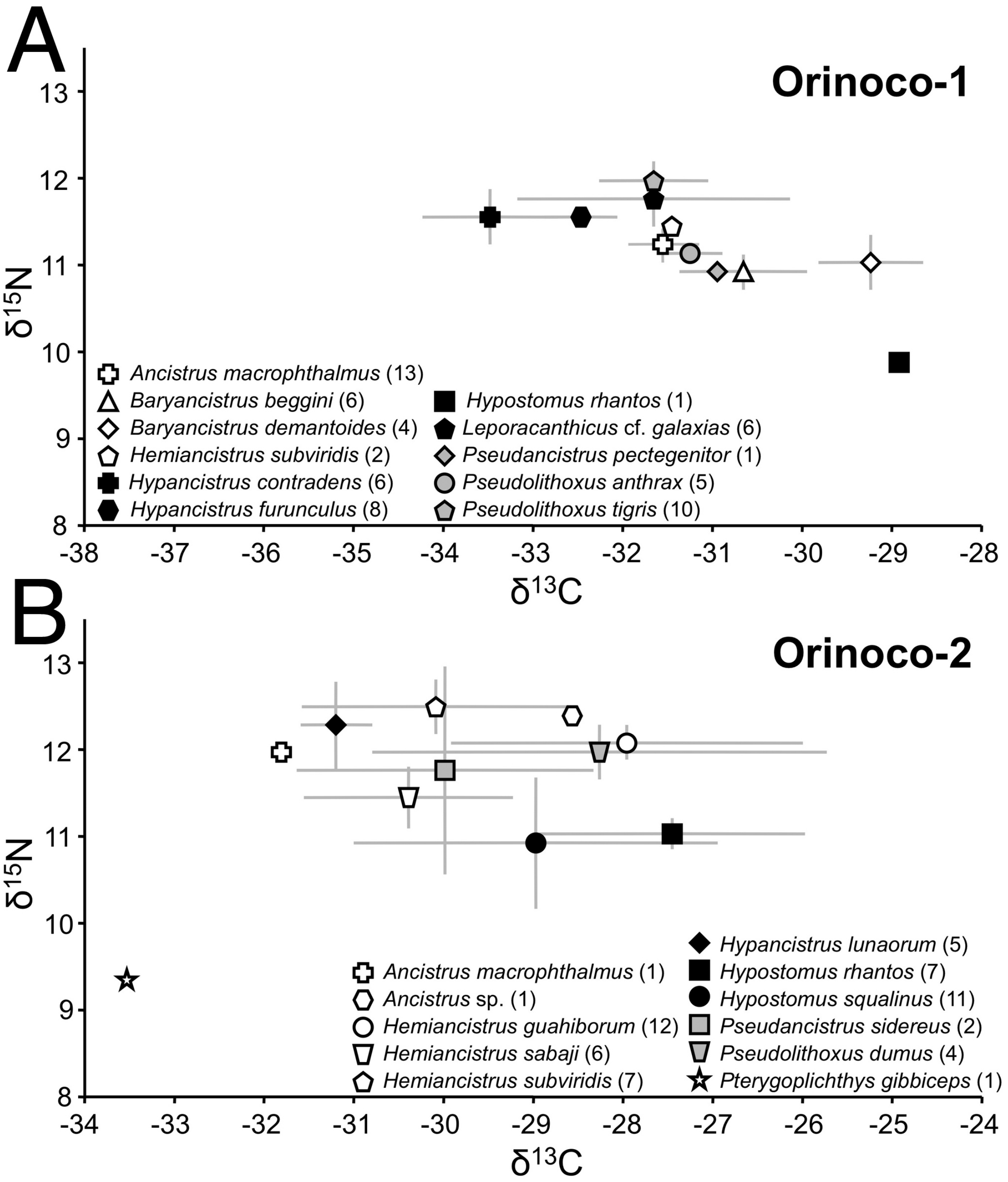

Figure 9 δ^{15}N and δ^{13}C relationships of loricariid consumers in (A) the Rupununi river, Essequibo drainage, Guyana; and (B) the Curuá river, Xingu drainage, Brazil. Sample sizes in parentheses.

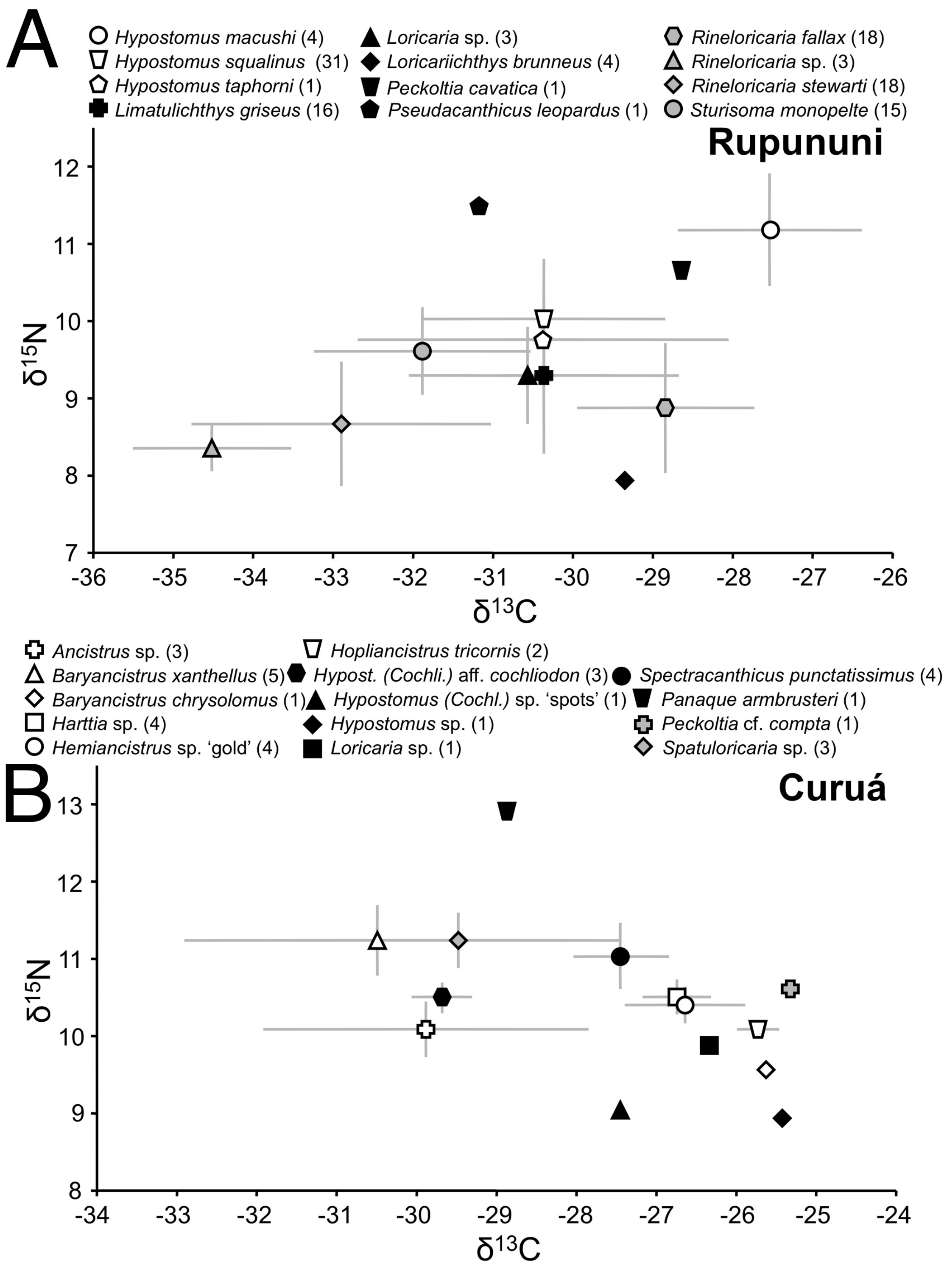

Figure 10 $\delta^{15}N$ and $\delta^{13}C$ relationships of loricariid consumers in (A) the Ventuari river, Orinoco drainage, southern Venezuela; and (B) the Marañon river, Amazon drainage, northern Peru. Sample sizes in parentheses.

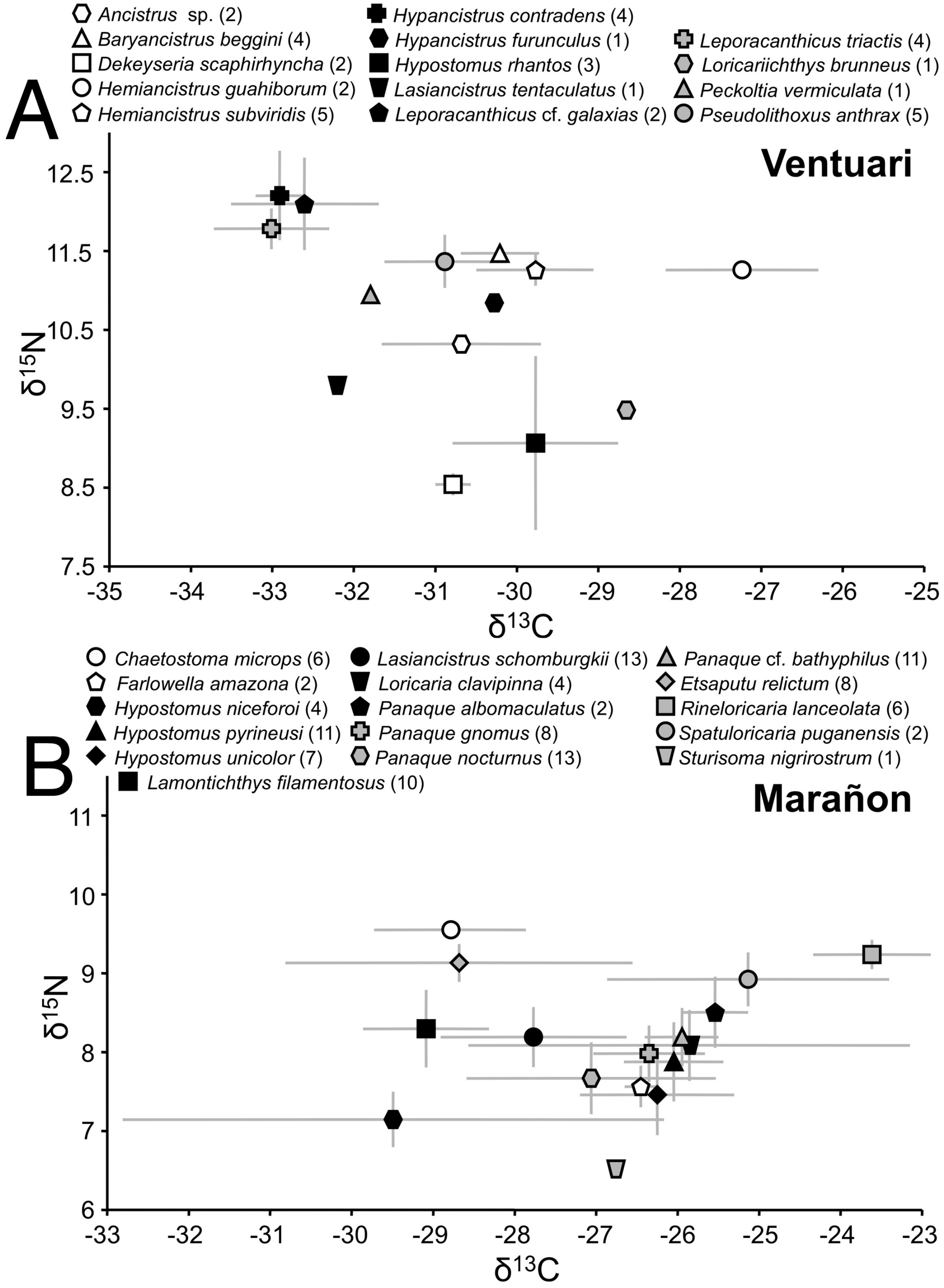

Supplement: Additional file 5 — Table S2. List of species by locality. [file 1471-2148-12-124-S5.pdf]
